# A novel C-terminal modification method enhanced the yield of human papillomavirus L1 or chimeric L1-L2 virus-like particles in the baculovirus system

**DOI:** 10.3389/fbioe.2022.1073892

**Published:** 2023-01-05

**Authors:** Mingrao Ma, Baicheng Xia, Zhirong Wang, Yaru Hao, Ting Zhang, Xuemei Xu

**Affiliations:** Department of Biophysics and Structural Biology, Institute of Basic Medical Sciences Chinese Academy of Medical Sciences, School of Basic Medicine Pecking Union Medical College, Beijing, China

**Keywords:** HPV, C-terminal substitution, L1 VLP, L1-L2 cVLP, yield, baculovirus system

## Abstract

Human papillomavirus (HPV) major capsid protein L1 virus-like particles (VLPs) produced in the baculovirus system showed excellent safety and immunogenicity, but the relatively high production cost stands as a substantial barrier to extensive commercialization, especially in producing multivalent vaccines. Here, a novel method, C-terminal basic amino acid (aa) substitution, was developed for increasing VLP and chimeric VLP (cVLP) production in this system. A series of mutants of five HPV types, including three L1 VLPs (6L1, 11L1, and 52L1) and two L1-L2 cVLPs (16L1-33L2, 58L1-16L2), were constructed. We found that most mutants exhibited higher protein expression in Sf9 cells, among which the yields of the superior mutants, 6L1CS4, 11L1CS3, 52L1m4∆N13CS1, 16L1-33L2 CS1, and 58L1-16L2 CS3, were up to 40, 35, 20, 35, and 60 mg/L, which respectively increased by 4.2-, 7.3-, 5-, 2.5-, and 3.4-fold, and they also showed robust immunogenicity and great stabilities. Additionally, we found that the increased level of steady-state mRNA may play a crucial role in promoting L1 protein expression. Our results demonstrated that this novel method was cost-effective and can be used to reduce the production costs of L1 VLPs and L1-L2 cVLPs to develop broadly protective and affordable multivalent HPV vaccines.

## 1 Introduction

Human papillomavirus (HPV), implicated in various benign and malignant lesions, has been identified almost 450 types thus far (McBride, 2022). High-risk types are the main carcinogen in the cervix, penis, vagina, anus, and oropharynx, among which HPV16,−18,−45,−33,−58,−52 are responsible for 86% of cervical cancers worldwide. The two low-risk types, HPV6 and HPV11, are barely connected with cancer but account for more than 90% of genital warts (ICO, 2021).

To date, five prophylactic HPV vaccines comprised of virus-like particles (VLPs) assembled from major capsid protein L1 have been licensed, including Cervarix (HPV16/18) produced in insect cells, Walrinvax (HPV16/18), Gardasil (HPV16/18/6/11) and Gardasil-9 (HPV16/18/6/11/31/33/45/52/58) produced in yeast, Cecolin (HPV16/18) produced in *Escherichia coli* (*E. coli*). They all can significantly decline vaccine-type HPV infections (Vesikari et al., 2015; Markowitz et al., 2018; Kreimer et al., 2020; Lei et al., 2020; Porras et al., 2020; Qiao et al., 2020; Tota et al., 2020; Whitworth et al., 2020; Basu et al., 2021). However, differences in their immunogenicity and efficacy were observed. Multiple clinical trials showed that Cervarix induced much higher peak and long-term neutralizing antibody levels against HPV16, HPV18, and tested non-vaccine types than Gardasil in women of different ages, although it contains only half a dose of HPV16 L1 VLPs and an equal dose of HPV18 L1 VLPs (Mariz et al., 2020; Mariz et al., 2021; Kann et al., 2021). Additionally, Cecolin did not show cross-protective efficacy, while it contains equal doses of HPV16 L1 VLPs and HPV18 L1 VLPs relative to Gardasil (Kudo et al., 2019; Qiao et al., 2020; Tota et al., 2020; Tsang et al., 2020). Their different immunogenicity and efficacy may be attributed to the different expression systems. Consistent with our speculation, a study in mice showed that HPV16 L1 VLPs produced in insect cells induced much higher neutralizing antibody titers than those produced in yeast in the absence of adjuvant (Kim et al., 2018). These data suggested that insect cells, a higher eukaryotic expression system, may be more conducive to generating highly immunogenic VLPs.

Improving VLP yields is a key priority for reducing HPV vaccine production costs which favors the expanded vaccination, especially in less developed regions where more than 80% of cervical cancer occurs (ICO, 2021). Early findings on naturally occurring variants of HPV16 L1 showed that even several residue changes could bring about significant differences in L1 protein expression levels and yields (Touze et al., 1998). However, residue changes occurring in the critical domain can also affect the assembly and immunogenicity of VLPs (Schädlich et al., 2009; Godi et al., 2019). Previous studies have already revealed the domain-function relationships of L1 proteins. Different HPV types share strikingly similar L1 core domains consisting of β-jelly rolls and α-helixes, where the protein sequences are relatively conserved. The prominent structural differences occur in the surface-exposed loops which connect the conserved regions and are the target for the majority of neutralizing antibodies (Buck et al., 2013; Roden and Stern, 2018). Therefore, sequence changes within the L1 core domains or loops may have a profound impact. The N- and C-terminal tails do not contain major neutralizing epitopes (Schädlich et al., 2009), the latter contains nuclear localization signal (NLS) sequences which consist of basic amino acid (aa) clusters and mediate the transport of L1 proteins into the nucleus (Zhou et al., 1991). Further research found that both the 5′- and 3′-end of L1 genes contain negative regulatory elements that could influence L1 protein expression (Kennedy et al., 1991; Collier et al., 2002; Ma et al., 2007). Thus, the researchers always introduced N- and/or C-terminal truncation to enhance L1 protein expression in different expression systems. For instance, In *E. coli*, the proper N-terminal truncations of HPV33, −45, −52, and −58 L1 could improve the soluble protein levels (Wei et al., 2018). In insect cells, the C-terminal truncations of HPV6, −16, and −58 L1 showed advantages in protein expression and purification (Müller et al., 1997; Xie et al., 2013; Sun et al., 2016), and parallel N- and C-terminal truncation of HPV58 L1 enhanced the protein expression and VLP yields (Wang et al., 2022).

However, these available strategies are ineffective for certain HPV types. In our hands, one such problematic type was HPV52, we found its yield was extremely low in the baculovirus system. Thus, multiple attempts were made accordingly. First, *52L1* gene was modified by codon optimization and C-terminal truncation (named *52L1∆C*), and its yield increased to 2 mg/L but was still very low. Then, *52L1∆C* was modified by residue substitution at natural high-frequency mutation sites. We found that mutating aspartic acid to glutamic acid at position 447 (D447E) could slightly increase protein expression, and the yield was still not satisfactory (4 mg/L). We subsequently constructed a series of N-terminally truncated mutants based on 52L1∆C containing the D447E mutation and unfortunately found that 13 aa deletion resulted in a significant increase in yield but a loss of immunogenicity. Clearly, the available strategies had limited success, and a novel method is required.

Here, we developed the C-terminal basic aa substitution method to enhance baculovirus-produced VLP yields of various HPV types. The basic aa located in NLS sequences were mutated to uncharged aa with small side chains or acidic aa, which could eliminate the negative elements located at the 3′-end of L1 genes, disrupt the nuclear localization signals, and allow the isoelectric point (pI) to change flexibly, thus favoring protein expression and purification. The mutants of five HPV types were constructed, including three L1 VLPs (6L1, 11L1, and 52L1) and two chimeric VLPs (cVLPs) displaying the conserved cross-neutralizing epitopes of minor capsid protein L2 (16L1-33L2, 58L1-16L2). We successfully obtained the mutants with robust immunogenicity and great stabilities, 6L1CS4, 11L1CS3, 52L1m4∆N13CS1, 16L1-33L2 CS1, and 58L1-16L2 CS3, whose yields were increased by 4.2-, 7.3-, 5-, 2.5- and 3.4-fold, and up to 40, 35, 20, 35, and 60 mg/L, respectively. Additionally, we have found that increased yields may be associated with increased steady-state mRNA levels and protein stabilities.

## 2 Materials and methods

### 2.1 Construction, expression, and purification of recombinant proteins

HPV*6* and HPV*11 L1* genes after codon-optimized for *S. frugiperda* (Sf9 cells) (Genbank accession no: OP379921 and OP379922, respectively) were synthesized by Sangon (Shanghai, China) and used as templates for polymerase chain reaction (PCR) to generate a series of C-terminal substitution constructs. Primers were listed in Supplementary Tables S1, S2, respectively. Then the PCR products were cloned into *Eco*RI/*Xba*lI sites in the pFastBac1 vector.

HPV*16L1-33L2* gene with codon optimization for Sf9 cells and C-terminal 31-residue truncation (Genbank accession no: OP379923) was used as a template to generate a series of C-terminal substitution constructs. Primers were listed in Supplementary Tables S7. Then the PCR products were cloned into *Bam*HI/*Eco*RI sites in the pFastBac1 vector.

HPV*52L1∆Cm4*, HPV*52L1∆Cm4∆N13*, and HPV*58L1-16L2* described in the supplementary methods were used as templates to generate HPV52 or HPV58 C-terminal substitution constructs which were cloned into *Bam*HI/*Eco*RI or *Bam*HI/*Xba*lI sites in the pFastBac1 vector, respectively. Primers were listed in Supplementary Tables S5, S6, and S8.

According to the Bac-to-Bac manufacturer’s instruction (Invitrogen), these recombinant plasmids were used to transform DH10Bac cells to generate recombinant bacmids, which were subsequently transfected into Sf9 cells to produce recombinant baculovirus and express recombinant proteins. To determine the optimal infection parameters to use for protein expression, we established a dose-response for each virus. Briefly, 1 × 10^6^ Sf9 cells were infected with recombinant baculovirus at varying MOIs (.1, 1, 2, 5, 8, 10, 12, 15, 18, and 20) and assay for protein expression. The MOIs that provided the optimal level of each recombinant protein expression were used to screen for the mutants with the highest expression levels.

All expressed proteins were purified by size-exclusion and cation-exchange chromatography. In brief, baculovirus-infected Sf9 cells were collected by centrifugation, resuspended in PBS, and lysed by sonication. The lysates were precipitated with 30% saturated (NH_4_)_2_SO_4_ for 2 h. Subsequently, the suspension was dialyzed for disassembly in 20 mM DTT-PBS at 4°C for 2 h, and then purified by two-step chromatography according to the manufacturer’s instructions (Cytiva). The fractions containing pure L1 or chimeric L1-L2 proteins were reassembled by dialyzing against .5 M NaCl-PBS, pH7.0 for 3–4 days. Protein concentrations were determined by the BCA Kit (Thermo Scientific).

### 2.2 SDS-PAGE and western blot analysis

Protein samples were mixed with the loading buffer containing 2% SDS and 5% β-mercaptoethanol and denatured at 75°C for 8 min. Denatured proteins were loaded onto 10% SDS-PAGE gels for separating and then transferred onto polyvinylidene difluoride membranes (PALL Life Sciences). The membranes were blocked with .05% Tween-80/PBS containing 5% non-fat milk at room temperature for 1–2 h and incubated with anti-HPV16 L1aa.230-236 monoclonal antibody Camvir-1 (dilution: 1/5000, Millipore Cat# MAB885) at 4°C overnight. Camvir-1 can be used to probe various HPV L1 proteins. Finally, horseradish peroxidase-conjugated secondary antibody (dilution: 1/5000, CWBio Cat# CW0102S) was added to react with Camvir-1 at room temperature for 1–2 h. The protein bands were revealed by chemiluminescence using the EasySee Western Bolt Kit (TransGen Biotech).

### 2.3 Dynamic light scattering (DLS) and transmission electron microscopy (TEM)

For DLS, protein samples were equilibrated to 25°C, and then the hydrodynamic diameter (Z-average) and polydispersity index (PdI) of particles were determined using Malvern Zatasizer Nano ZS90.

For TEM, protein samples at concentrations of 50–100 μg/ml were applied onto carbon-coated copper grids (Electron Microscopy China) and negatively stained with 2% uranyl acetate. Grids were analyzed using a TEM-1400 Plus electron microscope operating at 80 kV. Micrographs were captured at a magnification of 40,000-fold or 50,000-fold.

### 2.4 Animal immunization

BALB/c mice (female, 4–6 weeks) were purchased from SPF Biotechnology (Beijing, China) and kept in the animal facility of the Institute of Basic Medical Sciences, Chinese Academy of Medical Sciences. All animal studies followed the Institute of Laboratory Animal Science’s Institutional Animal Care and Use Committee’s guidelines and were approved by the Institutional Animal Care and Use Committee.

Mice (*n* = 5) were immunized subcutaneously either three times at 0, 4, and 8 weeks with 1 µg L1 VLPs alone or 10 µg L1-L2 cVLPs formulated with 50 µg Aluminium hydroxide gel (InvivoGen) and 5 µg monophosphoryl lipid A (MPLA) (InvivoGen), respectively. Sera were collected 2 weeks after the last boost and heat inactivated at 56°C for 30 min.

### 2.5 Pseudovirus (PsV) production and neutralization assay

Standard pseudoviruses (PsVs) were generated by co-infecting 293 TT cells with pCMV-Gluc 1 and HPV structural gene expression plasmids (p6SHELL, p11SHELL, p16SHELL, p52SHELL, or p58SHELL) following the online method (http://home.ccr.cancer.gov/Lco/pseudovirusproduction.htm). Furin-cleaved PsVs (fcPsVs) were generated by co-infecting 293TTF cells with pCMV-Gluc 1 and HPV structual gene expression plasmids (p16SHELL or p33SHELL) as Wang et al. (2014) described. Virus input doses were optimized as Nie et al. (2014) described.

Standard pseudovirus-based neutralization assay (PBNA) used to determine L1-raised neutralizing antibody titers was performed as previous studies (Nie et al., 2014). In brief, 293 TT cells were seeded in 96-well plates at the density of 3 × 10^4^ cells per well and incubated at 37°C and 5% CO_2_ overnight. The inactivated sera were two-fold serially diluted with Dulbecco’s modified Eagle’s medium (DMEM), starting at 1:1000. Subsequently, the serially diluted sera were mixed with equal volumes of PsV diluent, and the mixtures were incubated 4°C for 1 h. For negative controls, the mixtures were prepared by mixing equal volumes of PsV diluent and DMEM. Then, the above mixtures were added to the pre-plated cells and incubated at 37°C and 5% CO_2_. After 72 h, the Gluc activity was determined using Gaussia Luciferase Reporter Gene Assay Kit (Beyotime) and GloMax Navigator. The endpoint titers were calculated as the reciprocal of the highest sera dilutions with percent infection inhibition higher than 50%.

Furin-cleaved PBNA (FC-PBNA) used to determine L2-raised neutralizing antibody titers was performed as standard PBNA but with two modifications: LoVoT cells served as the target cell line instead of 293 TT cells; fcPsVs were used to infected cells instead of standard PsVs.

### 2.6 Quantitative real-time PCR

Total RNA was extracted from Sf9 cells infected with recombinant baculovirus at a multiplicity of infection (MOI) of five for 16 h using TRIzol (Invitrogen) following the manufacturer’s instruction. Extracted RNA was reverse transcribed to cDNA with oligo (dT) primers using the cDNA Synthesis Kit (CWBIO). Quantitative real-time PCR (RT-PCR) was undertaken using the UltraSYBR Mixture (CWBIO). Primer sequences for *52L1* designed by IDT PrimerQuest Tool (https://www.idtdna.com/pages/tools/primerquest) were 5′-GTC​CTC​AGG​AAA​CGG​TAA​GAA​G-3’ (sense) and 5′-TCT​CTG​GGT​CTC​TGG​GTT​ATA​G-3’ (antisense). Primer sequences for *GAPDH* (Genbank accession no: KT218670.1) used as the internal control were 5′-ACG​GAC​CCT​CTG​GAA​AAC​TG-3’ (sense) and 5′-GCA​ACG​GGA​ACA​CGG​AAA-3’ (antisense). The RT-PCR procedure was conducted as follows: 95°C for 10 min, 35 cycles of 94°C for 30 s, 58°C for 30 s, and 72°C for 20 s. The relative mRNA levels were calculated by the 2^−ΔΔCT^ method. The PCR products were detected by 1% agarose gel stained with CelRed nucleic acid dye (LABLEAD).

### 2.7 mRNA stability assay

For mRNA stability assessments, Sf9 cells infected with recombinant baculovirus at an MOI of five for 24 h were treated with 10 μg/ml actinomycin D (ActD) to block transcription. Total RNA was extracted at 0, .5, 1.0, and 1.5 h after ActD treatment, and the mRNA levels were determined by RT-PCR according to the above-described protocol.

### 2.8 Determination of the minimum free energy of mRNA secondary structures

The minimum free energy (MFE) of mRNA secondary structures was determined by Vienna RNAfold WebServer (http://rna.tbi.univie.ac.at/cgi-bin/RNAWebSuite/RNAfold.cgi). The complete RNA sequences were uploaded. The parameters were selected as follows: MFE and partition function; avoiding isolated base pairs; dangling energies on both sides of a helix in any case; RNA parameters (Turner model, 2004); energy parameters used in the calculation were measured at 37°C.

### 2.9 Statistical analysis

Statistical significance was determined by Student’s *t*-test (two-tailed, unpaired) using Graphpad Prism 8.0. *p* values were designated as follows: ns = not significant, *p* ≥ .05; *, *p* < .05; **, *p* < .01; ***, *p* < .001; ****, *p* < .0001.

## 3 Results

### 3.1 Comparison of the protein expression and yields of C-terminal substitution mutants in Sf9 cells

Our previous work showed that conventional gene modification strategies used to enhance protein expression have had limited success. We first synthesized *52L1* gene with codon optimization and C-terminal truncation (named *52L1∆C*), whose yield increased to 2 mg/L but was still very low. Then, we collected all naturally occurring 52L1 sequences from National Center for Biotechnology Information (NCBI) to find out specific mutations strongly related to protein expression. After sequence alignment, the top four high-frequency mutation sites, aa.184, aa.281, aa.357, and aa.447, were chosen. The most common substitutions that occurred at these sites were introduced into 52L1ΔC. As shown in Supplementary Figure S2, we have successfully found that mutating aspartic acid to glutamic acid at position 447 (52L1∆Cm4) could enhance L1 protein expression without compromising immunogenicity, but the yield was just 4 mg/L. Subsequently, a series of N-terminally truncated mutants based on 52L1∆Cm4 were constructed, among which 52L1∆Cm4∆N13 had a much higher yield (60 mg/L) but a loss of immunogenicity (Supplementary Figure S3).

**FIGURE 1:**
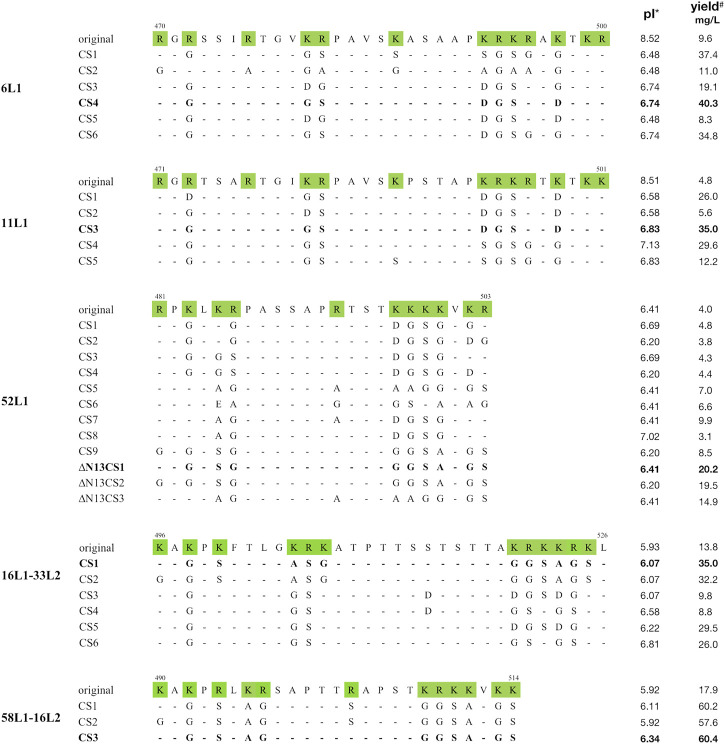
Alignment of C-terminal amino acid sequences of 6L1, 11L1, 52L1, 16L1-33L2, and 58L1-16L2. Amino acid numbers are shown on the top of each original sequence. Basic amino acids are shaded in green. Dashes indicate consensus amino acids compared to corresponding original sequences. The mutants with maximal yields of each type are bolded. *: Isoelectric point. ^#^: The mean yields of three biological repeats.

**FIGURE 2:**
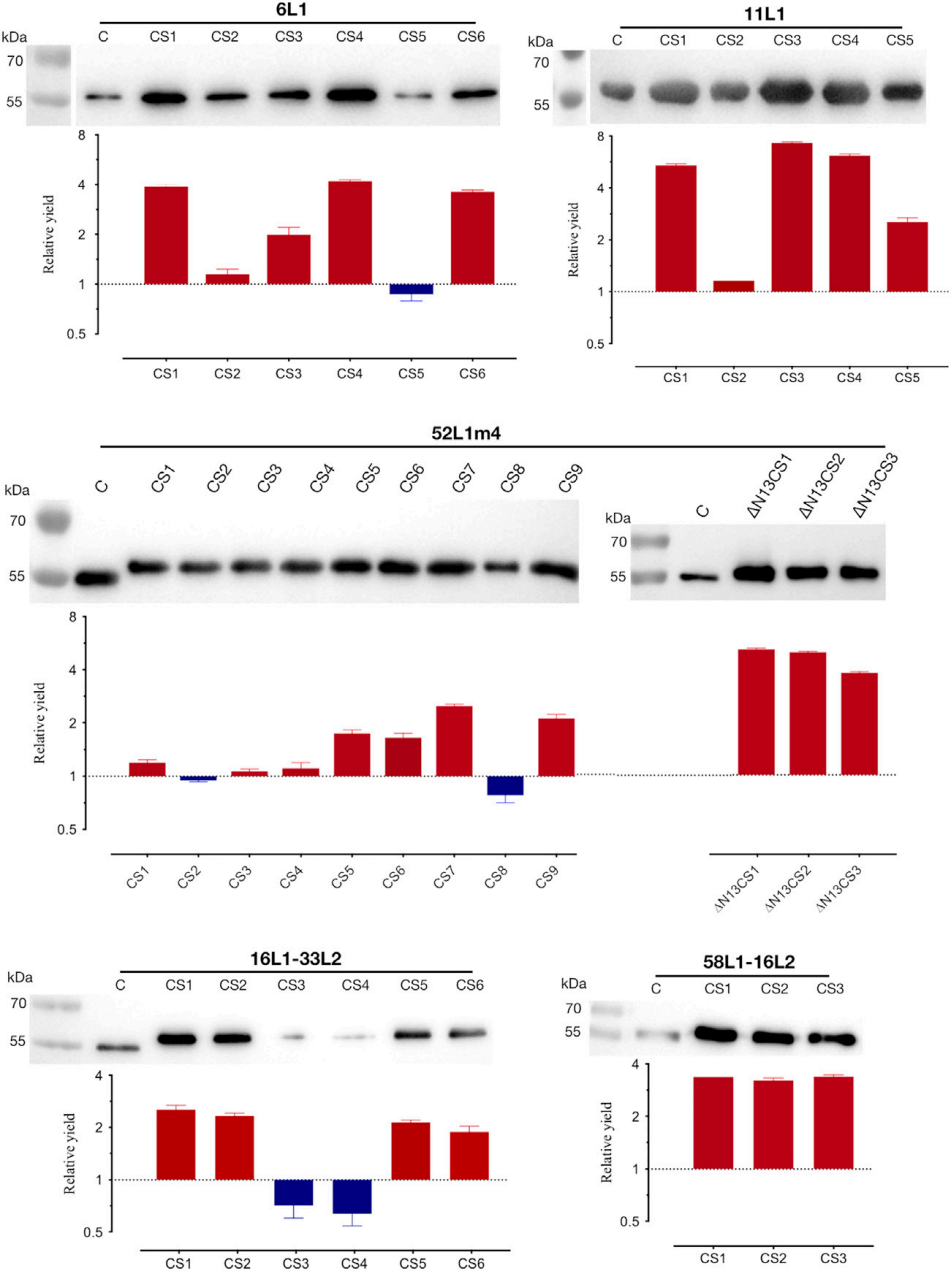
Analysis of protein expression and yields of the C-terminal substitutions of 6L1, 11L1, 52L1, 16L1-33L2, and 58L1-16L2. Each lane was loaded with 600 ng of whole-cell proteins for expression analysis by western blot, and L1 proteins were probed with mAb Camvir-1. Histograms depict fold-change in yields of purified proteins after C-terminal substitutions. The red and blue bars represent an increase or decrease in yields. Results represent three biological repeats. Data are presented as means±SD .

Thus, the C-terminal substitution method was developed, the rule of which was mutating basic aa located in the nuclear localization signal (NLS) domains to uncharged aa with small side chains or acidic amino acids. We constructed multiple mutants containing distinct patterns of basic aa substitutions in the C-terminus of 6L1, 11L1, 52L1, 16L1-33L2, or 58L1-16L2, and their C-terminal sequences were shown in Figure 1. Protein expression levels of these constructs were determined by SDS-PAGE (Supplementary Figure S1) and western blot (Figure 2) using Sf9 cell lysates. After substitution, most mutants showed higher expression levels, and the mutants with maximal expression were 6L1CS4, 11L1CS3, 52L1m4∆N13CS1, 16L1-33L2 CS1, and 58L1-16L2 CS3, respectively.

Subsequently, the yields of purified proteins were evaluated, which is the key factor in determining the production cost of vaccines. The highest protein yields of HPV6, −11, −52, −16, and −58 were up to 40 mg/L (6L1CS4), 35 mg/L (11L1CS3), 20 mg/L (52L1m4∆N13CS1), 35 mg/L (16L1-33L2 CS1) and 60 mg/L (58L1-16L2 CS3), which were 4.2-, 7.3-, 5-, 2.5- and 3.4-fold higher than that of proteins before C-terminal substitution of each type, respectively (Figure 2). Worth mentioning here that the yield of 52L1m4∆N13CS1 was increased by a factor of 10 compared to that of 52L1∆C (2 mg/L) we synthesized initially, although it was still slightly lower than other HPV types. Additionally, we found that the differences in protein yields were not completely consistent with the changes in expression levels. For instance, the protein expression levels of 52L1m4CS1, 52L1m4CS3, and 52L1m4CS4 were lower than that of 52L1∆Cm4, but their protein yields were slightly higher (Figure 2). Therefore, we speculate that C-terminal substitutions disrupted the NLS sequences and reduce the interaction between the protein and nuclear components, thus improving the recovery of protein purification.

### 3.2 Structure characterization of HPV6, −11, −52 L1 VLPs, and HPV16, −58 cVLPs

The C-terminal substitution mutants with the highest yields of each HPV type (6L1CS4, 11L1CS3, 52L1m4∆N13CS1, 16L1-33L2 CS1, and 58L1-16L2 CS3) were selected for further analysis. We used DLS and negative staining TEM to examine the assembly of L1 VLPs and L1-L2 cVLPs.

In DLS, the size distribution of all particles was unimodal, and the hydrodynamic diameter (particles with encircled layers of water molecules) of 6L1CS4, 11L1CS3, and 52L1m4∆N13CS1 VLPs were 79.04, 69.31, and 111.90 nm, of 16L1-33L2 CS1, and 58L1-16L2 CS3 cVLPs were 69.22, and 49.56 nm, respectively.

**FIGURE 3:**
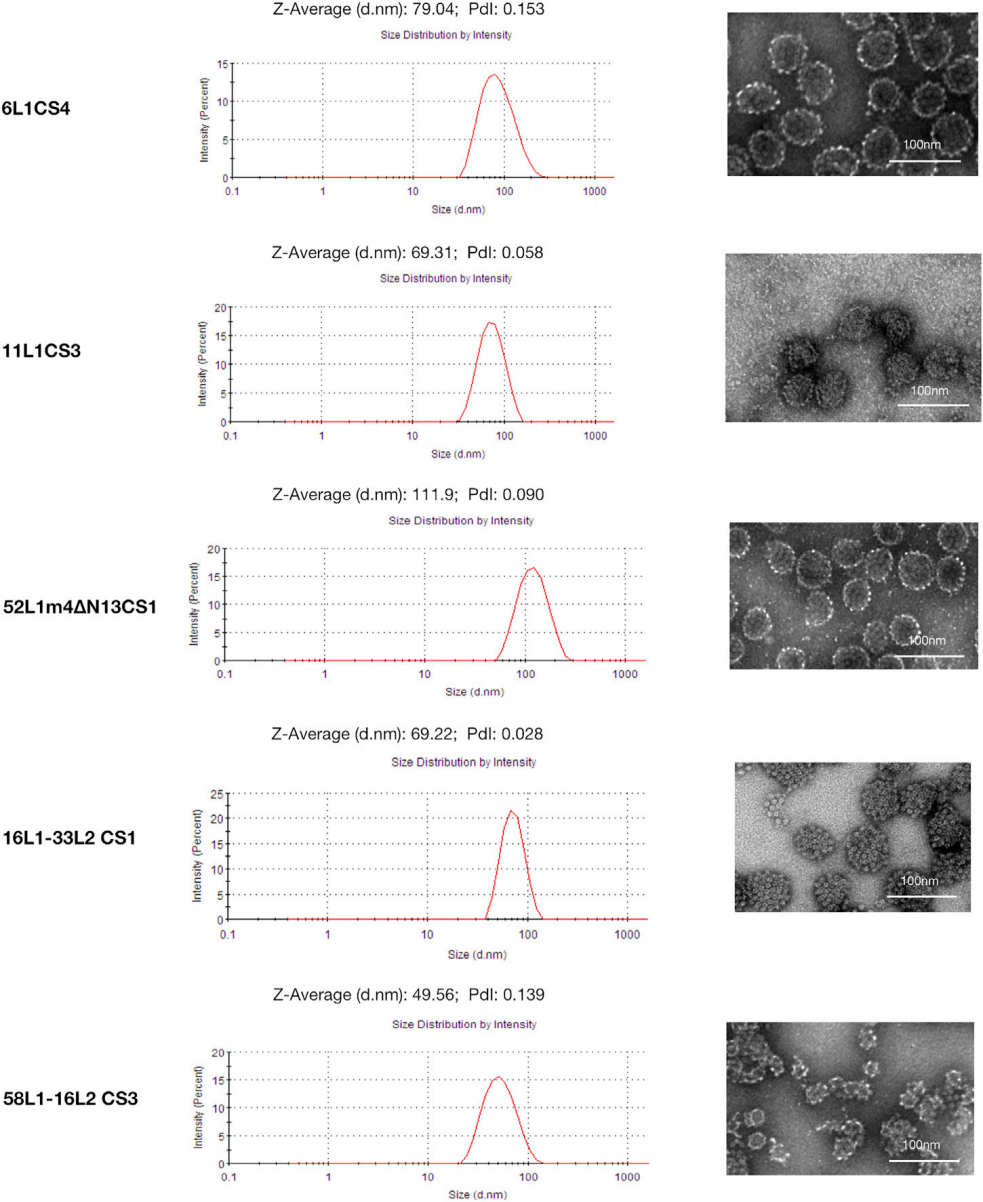
Dynamic light scattering (DLS) and transmission electron microscopy (TEM) analysis of 6L1CS4 VLPs, 11L1CS3 VLPs, 52L1m4ΔN13CS1 VLPs, 16L1-33L2 CS1 cVLPs, and 58L1-16L2 CS3 cVLPs. Purified proteins were analyzed by DLS and TEM with a magnification of ×40,000 (6L1CS4, 52L1m4ΔN13CS1, and 58L1-16L2 CS3) or 50,000 × (11L1CS3 and 16L1-33L2 CS1). Bar = 100 nm.

In TEM, the morphology of 6L1CS4 VLPs, 11L1CS3 VLPs, 52L1m4∆N13CS1 VLPs, and 16L1-33L2 CS1 cVLPs (diameters, ∼55 nm) was similar to that of VLPs reported before, while 58L1-16L2 CS3 cVLPs (diameter, ∼25 nm) appeared to be smaller than regular VLPs (Figure 3). It may be attributed to the different insertion sites for L2 epitopes. 16L1-33L2 and 58L1-16L2 cVLPs were constructed by inserting L2 epitopes within the DE loops or h4 coils of L1 proteins, respectively. The previous studies suggested that the h4-coil insertions may result in smaller-sized particles, such as 16L1-31L2 cVLPs (diameter, 25–48 nm) constructed by Chen et al. (2018), 18L1-33L2 and 18L1-58L2 cVLPs (diameters, 20–40 nm) constructed by Boxus et al. (2016). However, their type-specific immunogenicity was not affected. Thus we speculate that HPV58 cVLPs may also provide similar immunogenicity as regular-sized VLPs.

### 3.3 Analysis the stabilities of HPV6, −11, −52 L1 VLPs, and HPV16, −58 cVLPs.

**FIGURE 4:**
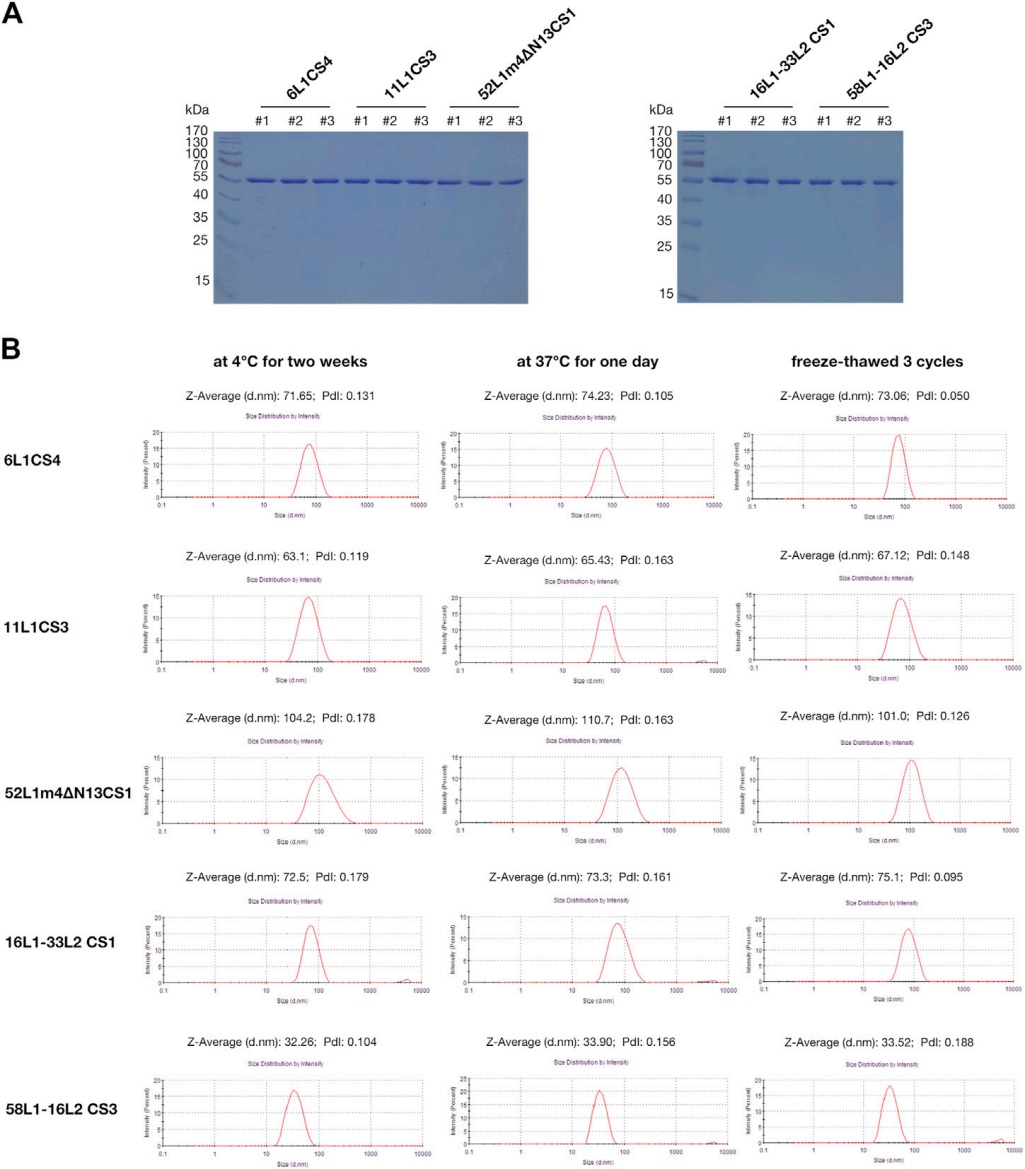
Analysis of the protein stabilities of 6L1CS4, 11L1CS3, 52L1m4∆N13CS1, 16L1-33L2 CS1, and 58L1-16L2 CS3 stored at 4 °C for 2 weeks, at 37°C for 1 day, or underwent three freeze-thaw cycles. **(A)** The protein degradation was analyzed by SDS-PAGE, and each lane was loaded with 5 μg of purified proteins. ^#^1: 4 °C for 2 weeks; ^#^2: 37°C for 1 day; ^#^3: Three freeze-thaw cycles. **(B)** The VLP stabilities were analyzed by DLS.

The stabilities of viral antigens are critical for vaccine production and transportation. We further evaluated the stabilities of the mutants under the following storage conditions by SDS-PAGE and DLS: maintenance at 4°C for 2 weeks, maintenance at 37°C for 1 day, and three freeze-thaw cycles. The SDS-PAGE results showed that all the proteins stored at these three conditions did not degrade (Figure 4A). In DLS, the hydrodynamic diameters of each HPV type under different storage conditions were stable, and the polydispersity index (PdI) values were all less than .2, which indicated that VLPs were monodisperse (Figure 4B). In addition, we selected a mutant with low expression (52L1m4CS4) and determined its stability by DLS, the results showed that freeze-thaw cycles resulted in protein agglutination, and PdI value was up to .602 (Supplementary Figure S4).

### 3.4 Analysis of the immunogenicity of HPV6, −11, −52 L1 VLPs, and HPV16, −58 cVLPs in mice

**FIGURE 5:**
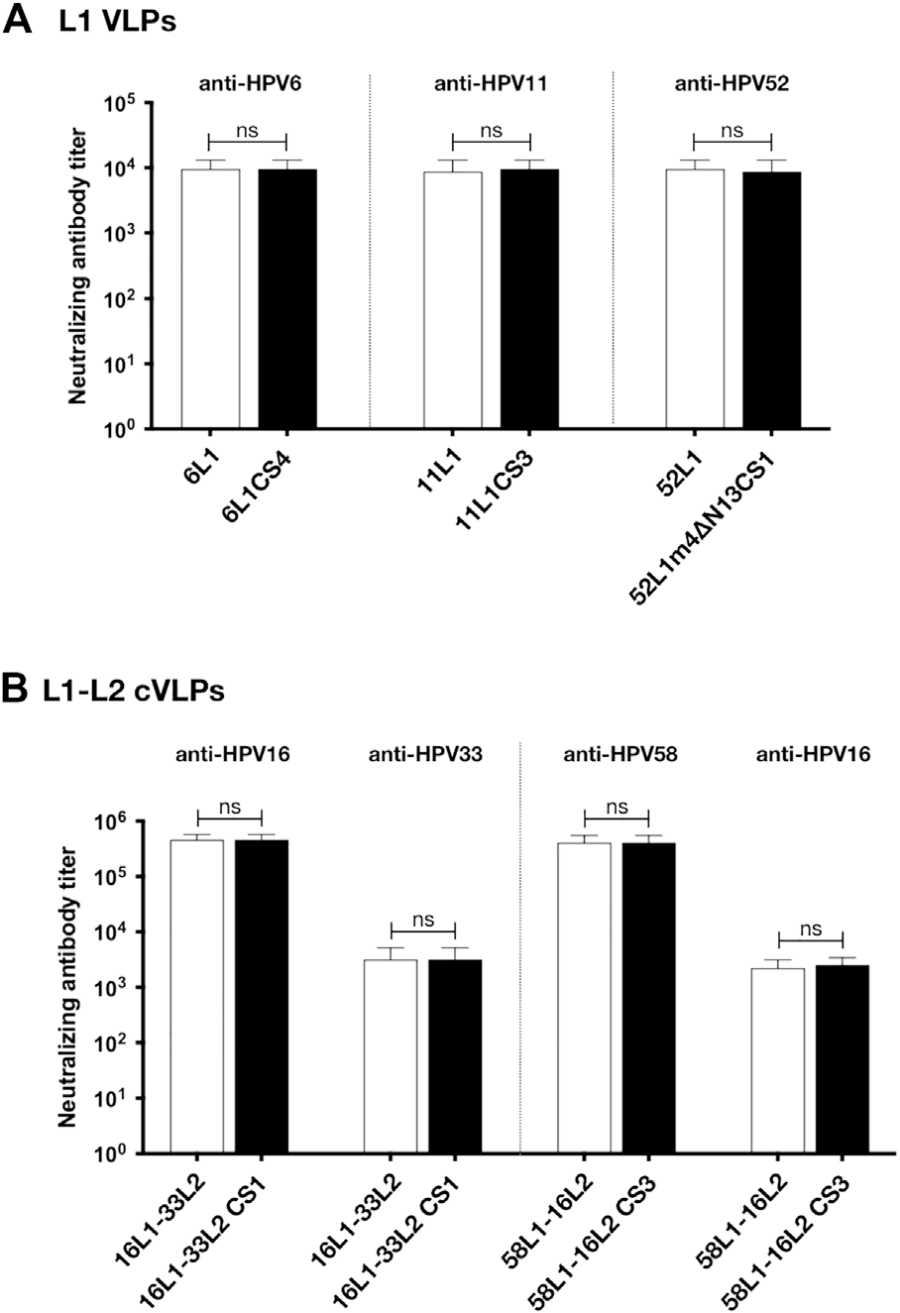
Immunogenicity analysis of 6L1CS4 VLPs, 11L1CS3 VLPs, 52L1m4ΔN13CS1 VLPs, 16L1-33L2 CS1 cVLPs, and 58L1-16L2 CS3 cVLPs in mice. At 0, 4, and 8 weeks, BALB/c mice (*n* = 5) were subcutaneously immunized with **(A)** 1 μg of HPV6, −11, or −52 L1 VLPs alone, **(B)** 10 μg of HPV16, or −58 cVLPs combined with 50 μg Alum and 5 μg MPLA. All sera were collected 2 weeks after the last immunization. L1-raised neutralizing antibodies were titrated by standard pseudovirus-based neutralization assay (PBNA), and L2-raised neutralizing antibodies by furin-cleaved PBNA (FC-PBNA). Data are presented as means±SD . The statistically significant difference (using Student’s *t*-test) is indicated by: ns, *p* ≥ .05.

To assess the viability of L1 VLPs and L1-L2 cVLPs that were constructed by C-terminal substitution as potential vaccine antigens, we next determined L1-raised neutralizing antibodies by standard pseudovirus-based neutralization assay (PBNA) and L2-raised neutralizing antibodies by furin-cleaved PBNA (FC-PBNA). The analysis of antibody levels elicited after a three-dose schedule revealed that the L1-specific neutralizing antibody titers induced by 1 μg of 6L1CS4, 11L1CS3, or 52L1m4∆N13CS1 VLPs were similar to those induced by the equal doses of 6L1, 11L1, or 52L1 VLPs, respectively (*p* values are >.99, .76, and .76, respectively). Previous studies showed that the L2-raised neutralizing antibody titers induced by L1-L2 cVLPs in mice were at least two orders of magnitude lower than L1-raised (Schellenbacher et al., 2009; Huber et al., 2015; Huber et al., 2017; Boxus et al., 2016; Chen et al., 2017; Chen et al., 2018). To enhance L2-raised immune responses, mice were immunized with the higher dosage (10 μg) of L1-L2 cVLPs adjuvanted with 50 µg Alum and 5 µg MPLA. The results showed that the anti-L1 and anti-L2 neutralizing antibody titers induced by 16L1-33L2 CS1 or 58L1-16L2 CS3 cVLPs were similar to those induced by the equivalent 16L1-33L2 (*p* values are >.99 and >.99, respectively) or 58L1-16L2 cVLPs, respectively (*p* values are >.99 and .58, respectively) (Figure 5).

Given that the mutants, 6L1CS4, 11L1CS3, 52L1m4∆N13CS1, 16L1-33L2 CS1, and 58L1-16L2 CS3, constructed by C-terminal substitution provided high yields and robust immunogenicity, they will be selected as antigens for our universal broad-spectrum vaccine formulation.

### 3.5 Analysis of L1 mRNA levels and stabilities

**FIGURE 6:**
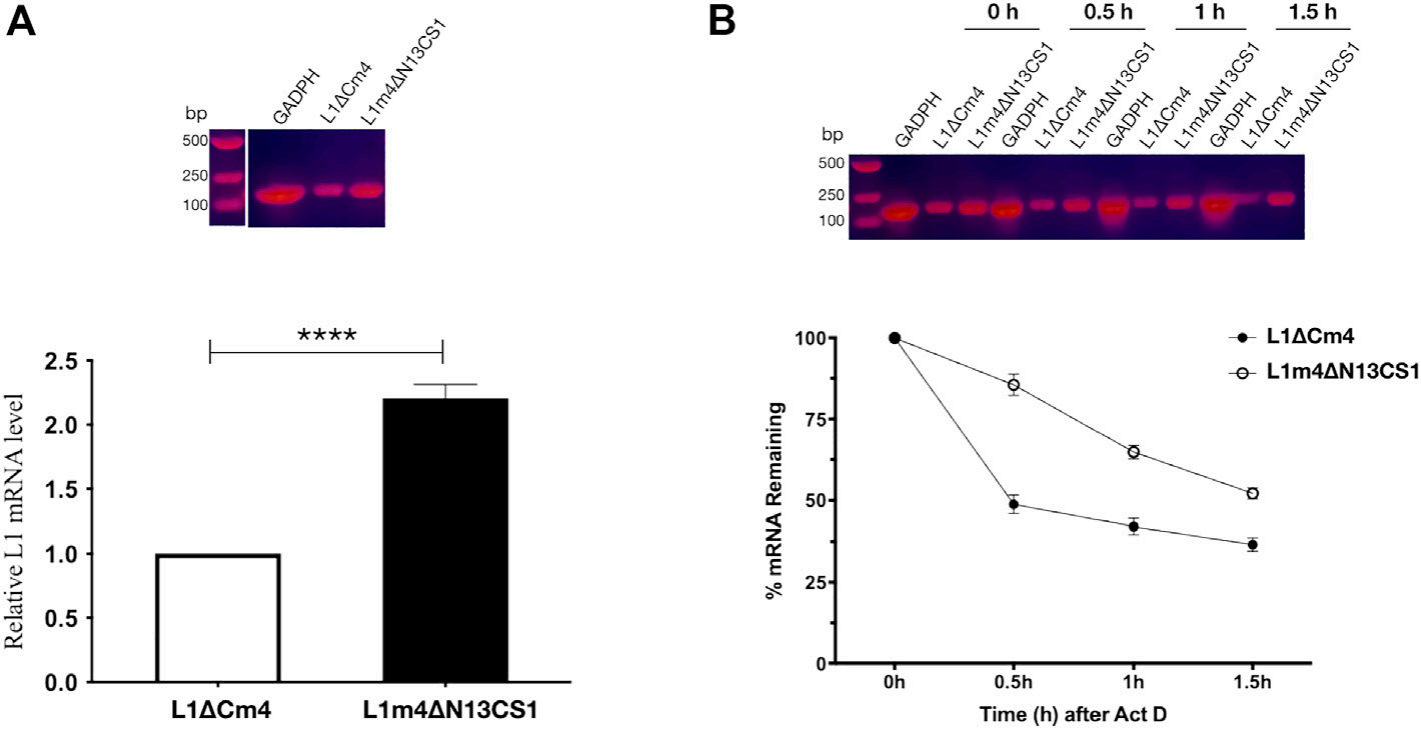
Analysis of 52L1∆Cm4 and 52L1m4∆N13CS1 mRNA levels and their stabilities by real-time PCR. **(A)** For mRNA level analysis, Sf9 cells infected with recombinant baculovirus containing *52L1∆Cm4* or *52L1m4∆N13CS1* genes were collected at 16 h post-infection. **(B)** For stability analysis, actinomycin D (ActD) (10 μg/ml) was added to inhibit transcription at 24 h post-infection, and total RNA was respectively extracted at 0, .5, 1.0, and 1.5 h after ActD treatment. The PCR products were detected by 1% agarose gel stained with CelRed nucleic acid dye. The relative mRNA amounts were calculated using the 2^−ΔΔCT^ method and normalized with internal control *GAPDH*. Results represent three biological repeats. Data are presented as means±SD . The statistically significant difference (using Student’s *t*-test) is indicated by: ****, *p* < .0001.

It has been noted that L1 VLP production can be significantly affected by L1 transcript quality and quantity (Neeper et al., 1996). Here we selected HPV52 to analyze whether the differences in expressed L1 amounts are attributable to differences in transcript levels, we extracted mRNA from Sf9 cells 16 h after the recombinant baculovirus infection and determined the amount of L1 transcripts using real-time PCR. In contrast, 2.2-fold higher abundant L1 transcripts were detected in cells infected with 52L1m4∆N13CS1 baculovirus than that in cells infected with 52L1∆Cm4 baculovirus (Figure 6A). Based on the observation of the different mRNA abundance, we further analyzed mRNA half-life using ActD, which binds DNA at the transcription initiation complex and inhibits the formation of novel mRNA. After 24 h infection of 52L1∆Cm4 or 52L1m4∆N13CS1 recombinant baculovirus, Sf9 cells were stimulated with ActD, and total RNA was isolated and quantified at different times. The results showed that 52L1m4∆N13CS1 mRNA also had a much lower decay rate after ActD treatment, 52L1∆Cm4 mRNA half-life was less than .5 h, while 52L1m4∆N13CS1 mRNA half-life was up to more than 1.5 h (Figure 6B). Additionally, we determined mRNA secondary structures of all five HPV types by Vienna RNAfold WebServer, and found that the mutants with C-terminal substitutions showed lower minimum free energy (MFE) (Supplementary Table S9) which signifies higher stabilities of mRNA secondary structures. Overall, these data indicated that C-terminal modification could enhance mRNA stability and abundance, and thus improve the protein expression level.

## 4 Discussion

The clinical trials of commercially available HPV vaccines indicated that VLPs produced in the baculovirus system showed excellent safety and immunogenicity (Mariz et al., 2020; Mariz et al., 2021; Kann et al., 2021). However, the relatively high production cost of this system stands as a substantial barrier to extensive commercialization, especially in producing multivalent vaccines. Improving the viral antigen yield can significantly reduce the production cost of vaccines. Here, we introduced a novel gene modification method, C-terminal basic aa substitution, to enhance the VLP yields. After systematically comparing the protein expression levels and yields among different C-terminal substitution versions of five HPV types, we found that most mutants showed higher expression levels and yields, among which the yields of the superior mutants, 6L1CS4, 11L1CS3, 52L1m4∆N13CS1, 16L1-33L2 CS1, and 58L1-16L2 CS3, were up to 40, 35, 20, 35 and 60 mg/L, which respectively increased by 4.2-, 7.3-, 5-, 2.5- and 3.4-fold (Figure 2). Worth mentioning here that all the control proteins have already been optimized by conventional gene modification methods as described in Materials and Methods and were expressed at higher levels than the corresponding wild-type sequences. Therefore, the yield advantages of C-terminal substitution mutants will be more significant if compared with the wild-type proteins. These results indicate that our novel C-terminal modification method can strongly enhance HPV L1 and chimeric L1-L2 protein expression and yields in the baculovirus system.

In addition to the excellent antigens of HPV vaccines, L1 VLPs are also the most commonly used carriers for displaying foreign epitopes to develop broad-spectrum cVLP vaccines (Schellenbacher et al., 2009; Huber et al., 2015; Huber et al., 2017; Boxus et al., 2016; Chen et al., 2017; Chen et al., 2018), which has aroused interest in developing cost-effective modification strategies for enhancing L1 VLP production. Wei et al. (2018) reported that N-terminal truncations of L1 proteins could affect their soluble expression levels in *E. coli*, and the expression-sensitive site on the N-terminus varied with HPV types. Several studies indicated that the C-terminally truncated L1 proteins might have advantages in expression and purification (Müller et al., 1997; Xie et al., 2013; Sun et al., 2016). Wang et al. (2022) found that combining proper N-terminal and C-terminal truncations could enhance HPV58 L1 protein expression in insect cells. Unfortunately, these conventional methods have proven to be inefficient in improving HPV52 L1 protein expression levels in the baculovirus system. Whereas, our C-terminal substitution method can significantly enhance its protein expression and yields in this system. Additionally, this novel method was also effective in improving the yields of the other two high-risk types and two low-risk types, indicating that it could be generally applied to various high- and low-risk types. More notably, this method exhibited a higher success rate for improving protein expression compared with the conventional methods (Figure 2; Supplementary Figures S2, S3), which may prove it cost-effective. Future efforts can further explore its applicability to cutaneous HPV.

It was reported that the C-terminal tail of HPV16 L1 contains the bipartite NLS sequences, which consist of basic aa clusters, one is KRKKRK (aa 525–530), another one is KRK (aa 510–512) and KR (aa 525–526). Other HPV L1 proteins possess similar C-terminal basic sequences (Zhou et al., 1991). The L1 proteins expressed in eukaryotic cells are synthesized in the cytoplasm and then migrate into the nucleus mediated by the NLS. Disrupting NLS can block the L1 protein from nuclear localization and reduce the ability of L1 VLPs to associate with the nuclear matrix and cellular DNA, which has potential benefits for protein extraction and purification (Zhou et al., 1991; Müller et al., 1997). Here, we disrupted the integrity of NLS sequences by basic aa substitution, allowing the net charge and hydrophobic/hydrophilic characteristic of L1 proteins to be flexibly altered (Figure 1), thus allowing proteins easier purified by chromatography.

There has been much debate about whether changes in mRNA level contribute substantially to protein-level regulation. Neeper et al. (1996) found that the increased HPV L1 transcripts could improve L1 protein expression and L1 VLP production in yeast, and the former changed more significantly, which may be due to mRNA decay. Thus, we first determined the contribution of mRNA amounts to protein expression in the baculovirus system and found that HPV52 L1 mRNA amount was increased 2.2-fold after C-terminal substitution (Figure 6A), which exhibited a positive correlation with protein expression and VLP yields. Then the stability of L1 mRNA was determined, as the mRNA decay rate directly affects the steady-state mRNA levels, thereby affecting protein synthesis. We found that HPV52 L1 mRNA half-life rose from .5 h to 1.5 h after C-terminal substitution (Figure 6B). Kennedy et al. (1991) have localized a negative regulatory element present in HPV16 genome around the position of the L1 stop codon, and the negative element acts to destabilize mRNA. Overall, the extended mRNA half-life may be attributed to negative element elimination by C-terminal mutation. These data indicate that steady-state mRNA levels greatly influence L1 protein expression.

Highly expressed proteins may tend to have excellent stabilities. For respiratory syncytial virus (RSV), middle east respiratory syndrome coronavirus (MERS-CoV), and severe acute respiratory syndrome coronavirus 2 (SARS-CoV-2), a key strategy of vaccine design was stabilizing the metastable prefusion conformation of fusion (F) or spike (S) glycoprotein which are important targets for antibody-mediated neutralization. To achieve this, aa substitutions were introduced to the critical domains for conformational change. After substitution, the proteins with higher expression levels and yields showed enhanced stabilities (Krarup et al., 2015; Pallesen et al., 2017; Hsieh et al., 2020; Wrapp et al., 2020). For HPV, L1 protein is the main target for neutralizing antibodies, we obtained the mutants with higher yields by substitutions of C-terminal basic aa of L1 proteins, the superior mutants showed the ability to withstand storage at 4°C for 2 weeks, at 37°C for 1 day, and three freeze-thaw cycles (Figure 4). However, the mutant with low expression (52L1m4CS4) showed poor stability after freeze-thaw cycles (Supplementary Figure S4), which indicates that proteins with higher expression may have greater stabilities.

In summary, we have developed a novel C-terminal modification method and successfully obtained 6L1CS4, 11L1CS3, 52L1m4∆N13CS1, 16L1-33L2 CS1, and 58L1-16L2 CS3 mutants with high yields, great stabilities, and robust immunogenicity, which will be promising candidates for developing broadly protective and affordable multivalent HPV vaccines. We hope this novel gene modification method will accelerate the industrial production of HPV vaccines to mitigate the public health burden and has broad implications for vaccine design.

## Data Availability

The original contributions presented in the study are included in the article/Supplementary Material, further inquiries can be directed to the corresponding authors.

## References

[B1] Basu P. Malvi S. G. Joshi S. Bhatla N. Muwonge R. Lucas E. (2021). Vaccine efficacy against persistent human papillomavirus (HPV) 16/18 infection at 10 years after one, two, and three doses of quadrivalent HPV vaccine in girls in India: A multicentre, prospective, cohort study. Lancet Oncol. 22, 1518–1529. 10.1016/S1470-2045(21)00453-8 34634254 PMC8560643

[B2] Boxus M. Fochesato M. Miseur A. Mertens E. Dendouga N. Brendle S. (2016). Broad cross-protection is induced in preclinical models by a human papillomavirus vaccine composed of L1/L2 chimeric virus-like particles. J. Virol. 90, 6314–6325. 10.1128/JVI.00449-16 27147749 PMC4936133

[B3] Buck C. B. Day P. M. Trus B. L. (2013). The papillomavirus major capsid protein L1. Virology 445, 169–174. 10.1016/j.virol.2013.05.038 23800545 PMC3783536

[B4] Chen X. Liu H. Wang Z. Wang S. Zhang T. Hu M. (2017). Human papillomavirus 16L1-58L2 chimeric virus-like particles elicit durable neutralizing antibody responses against a broadspectrum of human papillomavirus types. Oncotarget 8, 63333–63344. 10.18632/oncotarget.19327 28968993 PMC5609925

[B5] Chen X. Zhang T. Liu H. Hao Y. Liao G. Xu X. (2018). Displaying 31RG-1 peptide on the surface of HPV16 L1 by use of a human papillomavirus chimeric virus-like particle induces cross-neutralizing antibody responses in mice. Hum. Vaccines Immunother. 14, 2025–2033. 10.1080/21645515.2018.1464355 PMC6149973 29683766

[B6] Collier B. Oberg D. Zhao X. Schwartz S. (2002). Specific inactivation of inhibitory sequences in the 5’ end of the human papillomavirus type 16 L1 open reading frame results in production of high levels of L1 protein in human epithelial cells. J. Virol. 76, 2739–2752. 10.1128/jvi.76.6.2739-2752.2002 11861841 PMC135970

[B7] Godi A. Kemp T. J. Pinto L. A. Beddows S. (2019). Sensitivity of human papillomavirus (HPV) lineage and sublineage variant pseudoviruses to neutralization by nonavalent vaccine antibodies. J. Infect. Dis. 220, 1940–1945. 10.1093/infdis/jiz401 31412122 PMC6834066

[B8] Hsieh C. Goldsmith J. A. Schaub J. M. Divenere A. M. Kuo H. Javanmardi K. (2020). Structure-based design of prefusion-stabilized SARS-CoV-2 spikes. bioRxiv 0826, 1–9. 10.1101/2020.05.30.125484 PMC7402631 32703906

[B9] Huber B. Schellenbacher C. Jindra C. Fink D. Shafti-Keramat S. Kirnbauer R. (2015). A chimeric 18L1-45RG1 virus-like particle vaccine cross-protects against oncogenic alpha-7 human papillomavirus types. PLoS One 10, e0120152. 10.1371/journal.pone.0120152 25790098 PMC4366228

[B10] Huber B. Schellenbacher C. Shafti-Keramat S. Jindra C. Christensen N. Kirnbauer R. (2017). Chimeric L2-based virus-like particle (VLP) vaccines targeting cutaneous human papillomaviruses (HPV). PLoS One 12, e0169533. 10.1371/journal.pone.0169533 28056100 PMC5215943

[B11] ICO/IARC Information Centre on HPV and Cancer (HPV Information Centre) (2021). Human papillomavirus and related Diseases report. Avaialable at: https://hpvcentre.net/statistics/reports/XWX.pdf?t=1665284135951 (Accessed October 9, 2022).

[B12] Kann H. Lehtinen M. Eriksson T. Surcel H. M. Dillner J. Faust H. (2021). Sustained cross-reactive antibody responses after human papillomavirus vaccinations: Up to 12 Years Follow-up in the Finnish maternity Cohort. J. Infect. Dis. 223, 1992–2000. 10.1093/infdis/jiaa617 33009576

[B13] Kennedy I. M. Haddow J. K. Clements J. B. (1991). A negative regulatory element in the human papillomavirus type 16 genome acts at the level of late mRNA stability. J. Virol. 65, 2093–2097. 10.1128/jvi.65.4.2093-2097.1991 1848319 PMC240071

[B14] Kim H. J. Cho S. Y. Park M. H. Kim H. J. (2018). Comparison of the size distributions and immunogenicity of human papillomavirus type 16 L1 virus-like particles produced in insect and yeast cells. Arch. Pharm. Res. 41, 544–553. 10.1007/s12272-018-1024-4 29637494

[B15] Krarup A. Truan D. Furmanova-Hollenstein P. Bogaert L. Bouchier P. Bisschop I. J. M. (2015). A highly stable prefusion RSV F vaccine derived from structural analysis of the fusion mechanism. Nat. Commun. 6, 8143. 10.1038/ncomms9143 PMC4569726 26333350

[B16] Kreimer A. R. Sampson J. N. Porras C. Schiller J. T. Kemp T. Herrero R. (2020). Evaluation of durability of a single dose of the bivalent HPV vaccine: The CVT trial. J. Natl. Cancer Inst. 112, 1038–1046. 10.1093/jnci/djaa011 32091594 PMC7566548

[B17] Kudo R. Yamaguchi M. Sekine M. Adachi S. Ueda Y. Miyagi E. (2019). Bivalent human papillomavirus vaccine effectiveness in a Japanese population: High vaccine-type-specific effectiveness and evidence of cross-protection. J. Infect. Dis. 219, 382–390. 10.1093/infdis/jiy516 30299519 PMC6325350

[B18] Lei J. Ploner A. Elfström K. M. Wang J. Roth A. Fang F. (2020). HPV vaccination and the risk of invasive cervical cancer. N. Engl. J. Med. 383, 1340–1348. 10.1056/nejmoa1917338 32997908

[B19] Ma Z. Chen B. Zhang F. Yu M. Liu T. Liu L. (2007). Increasing the expression levels of papillomavirus major capsid protein in *Escherichia coli* by N-terminal deletion. Protein Expr. Purif. 56, 72–79. 10.1016/j.pep.2007.05.010 17616400

[B20] Mariz F. C. Bender N. Anantharaman D. Basu P. Bhatla N. Pillai M. R. (2020). Peak neutralizing and cross-neutralizing antibody levels to human papillomavirus types 6/16/18/31/33/45/52/58 induced by bivalent and quadrivalent HPV vaccines. npj Vaccines 5, 14–21. 10.1038/s41541-020-0165-x 32128255 PMC7021830

[B21] Mariz F. C. Gray P. Bender N. Eriksson T. Kann H. Apter D. (2021). Sustainability of neutralising antibodies induced by bivalent or quadrivalent HPV vaccines and correlation with efficacy: A combined follow-up analysis of data from two randomised, double-blind, multicentre, phase 3 trials. Lancet Infect. Dis. 21, 1458–1468. 10.1016/S1473-3099(20)30873-2 34081923

[B22] Markowitz L. E. Drolet M. Perez N. Jit M. Brisson M. (2018). Human papillomavirus vaccine effectiveness by number of doses: Systematic review of data from national immunization programs. Vaccine 36, 4806–4815. 10.1016/j.vaccine.2018.01.057 29802000

[B23] McBride A. A. (2022). Human papillomaviruses: Diversity, infection and host interactions. Nat. Rev. Microbiol. 20, 95–108. 10.1038/s41579-021-00617-5 34522050

[B24] Müller M. Zhou J. Reed T. D. Rittmüller C. Burger A. Gabelsberger J. (1997). Chimeric papillomavirus-like particles. Virology 234, 93–111. 10.1006/viro.1997.8591 9234950

[B25] Neeper M. P. Hofmann K. J. Jansen K. U. (1996). Expression of the major capsid protein of human papillomavirus type 11 in Saccharomyces cerevisae. Gene 180, 1–6. 10.1016/S0378-1119(96)00388-5 8973339

[B26] Nie J. Huang W. Wu X. Wang Y. (2014). Optimization and validation of a high throughput method for detecting neutralizing antibodies against human papillomavirus (HPV) based on pseudovirons. J. Med. Virol. 86, 1542–1555. 10.1002/jmv.23995 24895216

[B27] Pallesen J. Wang N. Corbett K. S. Wrapp D. Kirchdoerfer R. N. Turner H. L. (2017). Immunogenicity and structures of a rationally designed prefusion MERS-CoV spike antigen. Proc. Natl. Acad. Sci. U. S. A. 114, E7348–E7357. 10.1073/pnas.1707304114 28807998 PMC5584442

[B28] Porras C. Tsang S. H. Herrero R. Guillén D. Darragh T. M. Stoler M. H. (2020). Efficacy of the bivalent HPV vaccine against HPV 16/18-associated precancer: Long-term follow-up results from the Costa Rica vaccine trial. Lancet Oncol. 21, 1643–1652. 10.1016/S1470-2045(20)30524-6 33271093 PMC8724969

[B29] Qiao Y. L. Wu T. Li R. C. Hu Y. M. Wei L. H. Li C. G. (2020). Efficacy, safety, and immunogenicity of an Escherichia coli-produced bivalent human papillomavirus vaccine: An interim analysis of a randomized clinical trial. J. Natl. Cancer Inst. 112, 145–153. 10.1093/jnci/djz074 31086947 PMC7019098

[B30] Roden R. B. S. Stern P. L. (2018). Opportunities and challenges for human papillomavirus vaccination in cancer. Nat. Rev. Cancer 18, 240–254. 10.1038/nrc.2018.13 29497146 PMC6454884

[B31] Schädlich L. Senger T. Gerlach B. Mücke N. Klein C. Bravo I. G. (2009). Analysis of modified human papillomavirus type 16 L1 capsomeres: The ability to assemble into larger particles correlates with higher immunogenicity. J. Virol. 83, 7690–7705. 10.1128/jvi.02588-08 19457985 PMC2708645

[B32] Schellenbacher C. Roden R. Kirnbauer R. (2009). Chimeric L1-L2 virus-like particles as potential broad-spectrum human papillomavirus vaccines. J. Virol. 83, 10085–10095. 10.1128/jvi.01088-09 19640991 PMC2748020

[B33] Sun B. Zhao D. Zhang X. Gu T. Yu X. H. Sun S. (2016). Development a scalable production process for truncated human papillomavirus type-6 L1 protein using WAVE Bioreactor and hollow fiber membrane. Appl. Microbiol. Biotechnol. 100, 1231–1240. 10.1007/s00253-015-6974-6 26446387

[B34] Tota J. E. Struyf F. Sampson J. N. Gonzalez P. Ryser M. Herrero R. (2020). Efficacy of the AS04-Adjuvanted HPV16/18 vaccine: Pooled analysis of the Costa Rica vaccine and PATRICIA randomized controlled trials. J. Natl. Cancer Inst. 112, 818–828. 10.1093/jnci/djz222 31697384 PMC7825474

[B35] Touze A. El Mehdaoui S. Sizaret P. Y. Mougin C. Muñoz N. Coursaget P. (1998). The L1 major capsid protein of human papillomavirus type 16 variants affects yield of virus-like particles produced in an insect cell expression system. J. Clin. Microbiol. 36, 2046–2051. 10.1128/JCM.36.7.2046-2051.1998 9650960 PMC104976

[B36] Tsang S. H. Sampson J. N. Schussler J. Porras C. Wagner S. Boland J. (2020). Durability of cross-protection by different schedules of the bivalent HPV vaccine: The CVT Trial. J. Natl. Cancer Inst. 112, 1030–1037. 10.1093/jnci/djaa010 32091596 PMC7566371

[B37] Vesikari T. Brodszki N. Van Damme P. Diez-Domingo J. Icardi G. Petersen L. K. (2015). A randomized, double-blind, phase III study of the immunogenicity and safety of a 9-valent human papillomavirus L1 virus-like particle vaccine (V503) versus Gardasil® in 9-15-year-old girls. Pediatr. Infect. Dis. J. 34, 992–998. 10.1097/INF.0000000000000773 26090572

[B38] Wang J. W. Jagu S. Kwak K. Wang C. Peng S. Kirnbauer R. (2014). Preparation and properties of a papillomavirus infectious intermediate and its utility for neutralization studies. Virology 449, 304–316. 10.1016/j.virol.2013.10.038 24418565 PMC3932537

[B39] Wang Z. Zhang T. Xu X. (2022). Combined truncations at both N- and C-terminus of human papillomavirus type 58 L1 enhanced the yield of virus-like particles produced in a baculovirus system. J. Virol. Methods 301, 114403. 10.1016/j.jviromet.2021.114403 34890711

[B40] Wei M. Wang D. Li Z. Song S. Kong X. Mo X. (2018). N-terminal truncations on L1 proteins of human papillomaviruses promote their soluble expression in *Escherichia coli* and self-assembly *in vitro* . Emerg. Microbes Infect. 7, 1–12. 10.1038/s41426-018-0158-2 30254257 PMC6156512

[B41] Whitworth H. S. Gallagher K. E. Howard N. Mounier-Jack S. Mbwanji G. Kreimer A. R. (2020). Efficacy and immunogenicity of a single dose of human papillomavirus vaccine compared to no vaccination or standard three and two-dose vaccination regimens: A systematic review of evidence from clinical trials. Vaccine 38, 1302–1314. 10.1016/j.vaccine.2019.12.017 31870572

[B42] Wrapp D. Wang N. Corbett K. S. Goldsmith J. A. Hsieh C. L. Abiona O. (2020). Cryo-EM structure of the 2019-nCoV spike in the prefusion conformation. Science 367, 1260–1263. 10.1126/science.abb2507 32075877 PMC7164637

[B43] Xie X. Liu Y. Zhang T. Xu Y. Bao Q. Chen X. (2013). Human papillomavirus type 58 L1 virus-like particles purified by two-step chromatography elicit high levels of long-lasting neutralizing antibodies. Arch. Virol. 158, 193–199. 10.1007/s00705-012-1465-x 22965579

[B44] Zhou J. Doorbar J. Sun X. Y. Crawford L. V. McLean C. S. Frazer I. H. (1991). Identification of the nuclear localization signal of human papillomavirus type 16 L1 protein. Virology 185, 625–632. 10.1016/0042-6822(91)90533-H 1660197

