# Supplementary material for: A novel C-terminal modification method enhanced the yield of human papillomavirus L1 or chimeric L1-L2 virus-like particles in the baculovirus system
Source: Front Bioeng Biotechnol. 2023 Jan 5;10:1073892. doi: 10.3389/fbioe.2022.1073892 (PMC9849392; doi:10.3389/fbioe.2022.1073892)
Supplement: Supplementary file 1 [file DataSheet3.docx]

Supplementary Material

# Supplementary Materials and Methods

## Construction of *58L1-16L2* gene

Based on our previous work, 58L1ΔN4C mutant with combined truncations at the N- and C-terminus of L1 protein was expressed at a high level in the baculovirus system (Wang et al., 2022) and used as the backbone to constructed 58L1-16L2 chimeric virus-like particles (VLPs) by displaying 16L2aa.17-38 on the surface loops (unpublished data).

## Construction of *52L1* genes

*52L1ΔC* gene with codon optimization for *S. frugiperda* (Sf9 cells) and C-terminal 19-residue truncation (Genbank accession no.: OP379920) was synthesized by Sangon (Shanghai, China) and shown to be expressed at a higher level in Sf9 cells (data not show). Then, a collection of 129 naturally occurring 52L1 sequences from National Center for Biotechnology Information was chosen and aligned similarly. The top four high-frequency mutation sites were aa.184, aa.281, aa.357, and aa.447, and the most common mutations that occurred at these sites were N184T (mutation frequency, 43%), Q281K (100%), S357D (89%), and D447E (100%). These four hot substitutions were introduced into *52L1ΔC* by overlap PCR and generated the mutants containing N184T, Q281K, S357D, D447E, Q281K/S357D/D447E, or N184T/Q281K/S357D/D447E mutations (successively named *52L1ΔCm1* to *52L1ΔCm6*). Primers were listed in **Table S3**.

*52L1ΔCm4* gene was further used to construct N-terminal truncation mutants by PCR, the removal residues were aa.2 (S), aa.2-4 (SVW), aa.2-5 (SVWR), aa.2-8 (SVWRPSE), aa.2-10 (SVWRPSEAT), aa.2-13 (SVWRPSEATVYL), aa.2-15 (SVWRPSEATVYLPP), aa.2-18 (SVWRPSEATVYLPPVPV), or aa.2-20 (SVWRPSEATVYLPPVPVSK), respectively. Primers were listed in **Table S4**.

# Supplementary Figures and Tables

## Supplementary Figures


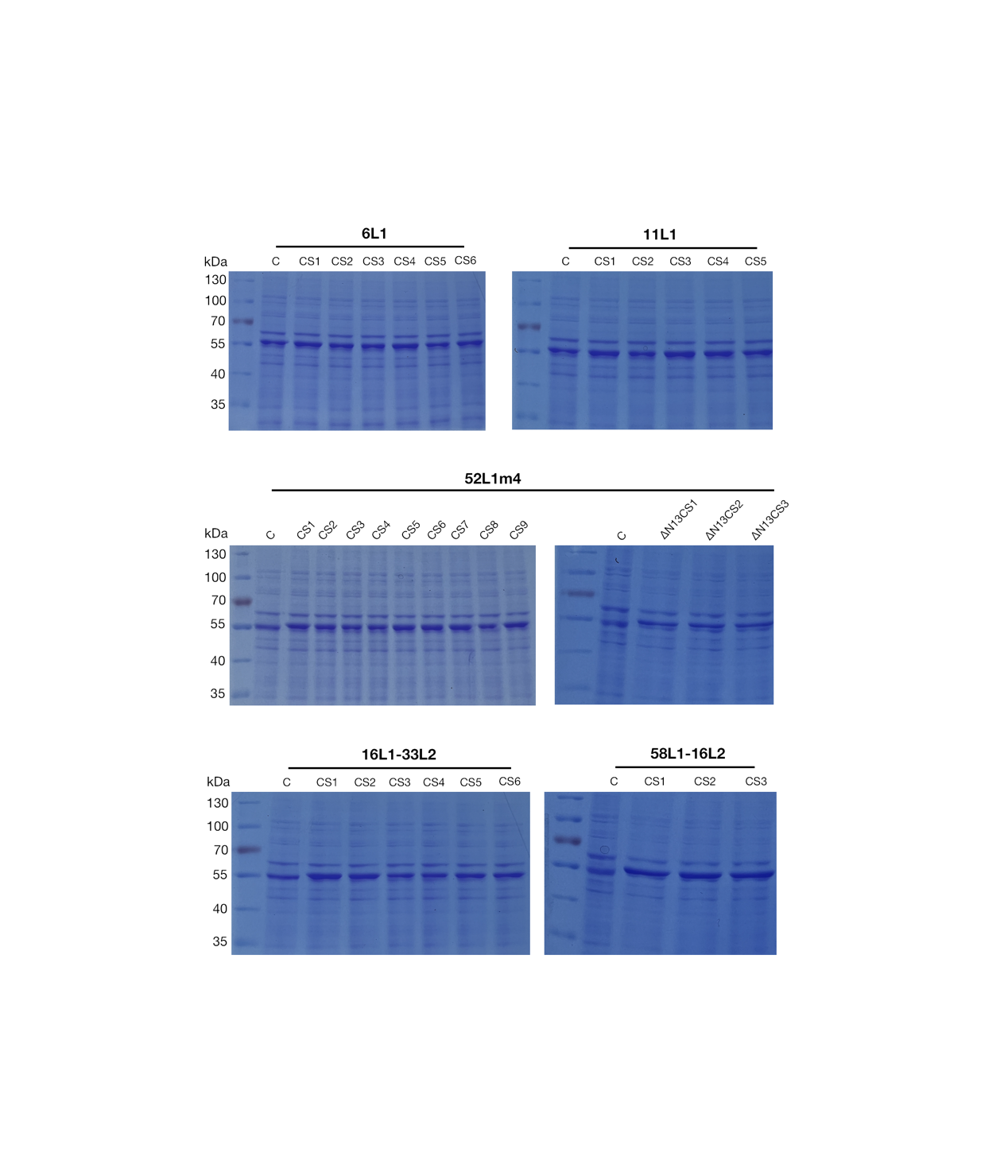


**Figure S1.** Expression analysis of C-terminal substitutions of 6L1, 11L1, 52L1, 16L1-33L2, and 58L1-16L2 in Sf9 cells by SDS-PAGE. Each lane was loaded with 15 μg of whole-cell proteins.


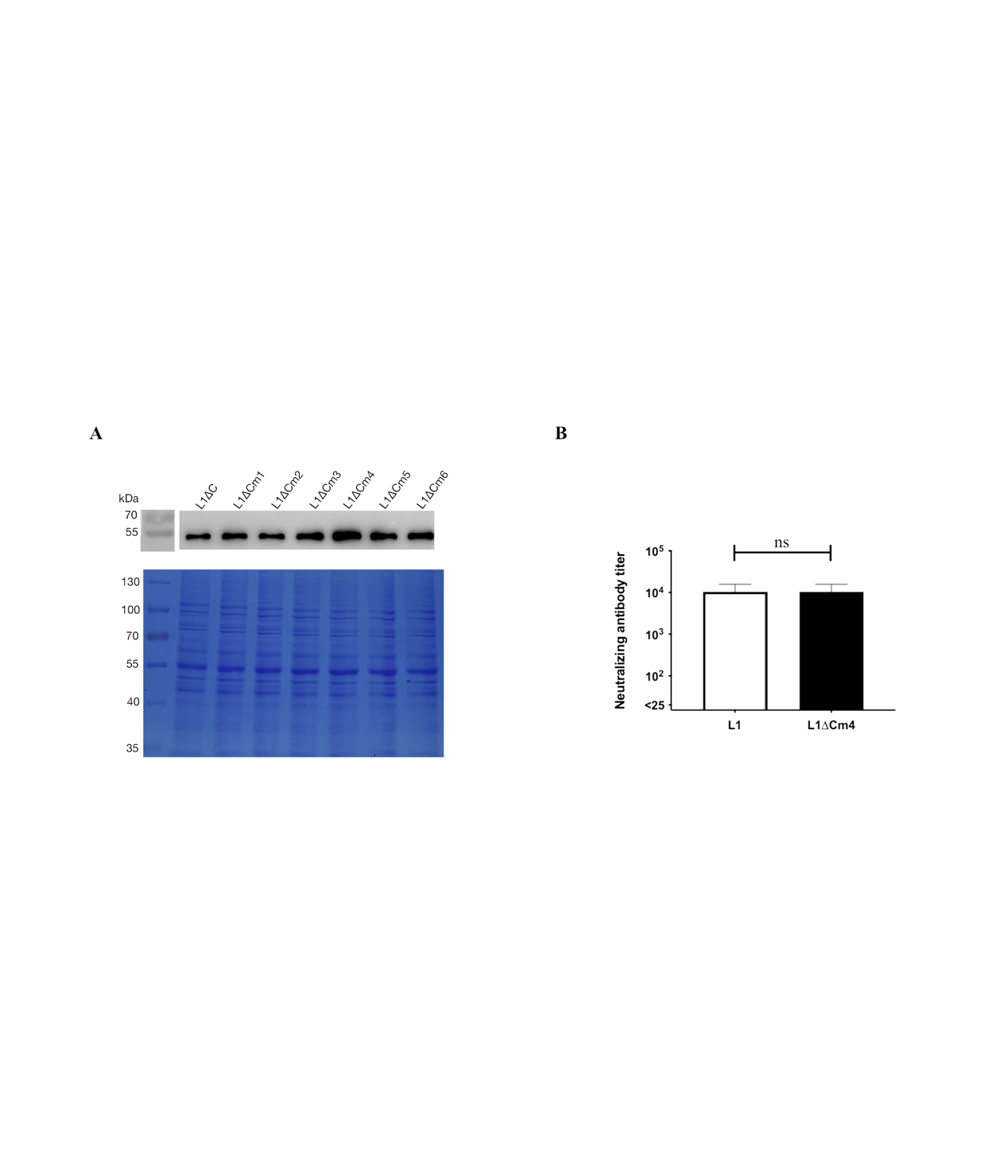


**Figure S2.** Expression and immunogenicity analysis of point mutated 52L1∆C proteins. **(A)** Expression analysis of point mutated 52L1∆C proteins in Sf9 cells. Whole-cell lysates were analyzed by SDS-PAGE (15 μg/lane) and western blot (600 ng/lane). L1 proteins were probed with mAb Camvir-1. **(B)** Immunogenicity analysis of 52L1∆Cm4 VLPs in mice. BALB/c mice (n = 5) were immunized subcutaneously with 1 μg of VLPs at 0, 4, 8 weeks. Sera were collected two weeks after the last immunization and titrated using a standard pseudovirion-based neutralization assay. Data are presented as $means\pm SD$. The statistically significant difference (using Student’s *t*-test) is indicated by: ns, *P*$\geq$0.05.


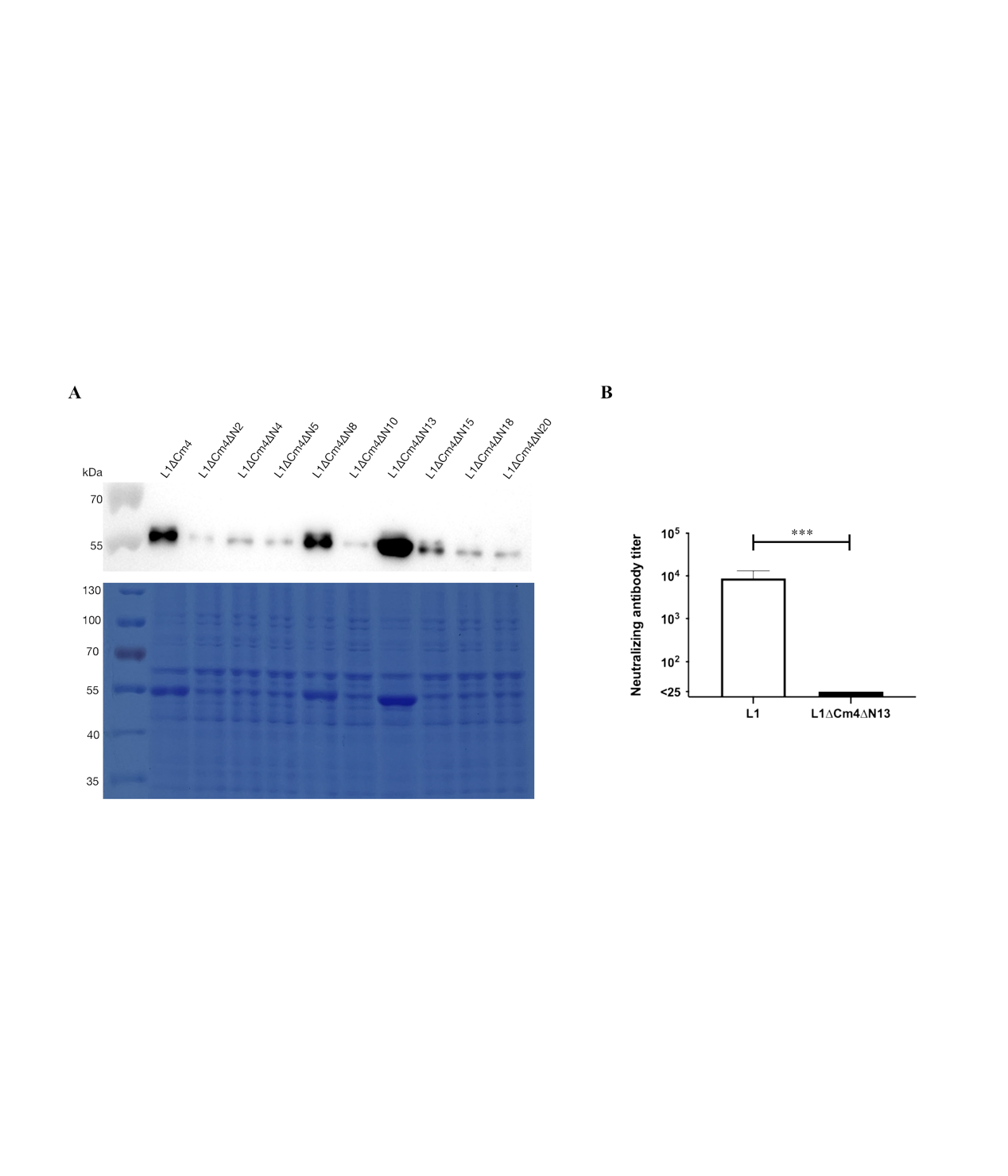


**Figure S3.** Expression and immunogenicity analysis of N-terminally truncated 52L1∆Cm4 proteins. **(A)** Expression analysis of N-terminally truncated 52L1∆Cm4 proteins in Sf9 cells. Whole-cell lysates were analyzed by SDS-PAGE (15 μg/lane) and western blot (600 ng/lane). L1 proteins were probed with mAb Camvir-1. **(B)** Immunogenicity analysis of 52L1∆Cm4∆N13 VLPs in mice. BALB/c mice (n = 5) were immunized subcutaneously with 1 μg of VLPs at 0, 4, 8 weeks. Sera were collected two weeks after the last immunization and titrated using a standard pseudovirion-based neutralization assay. Data are presented as $\mathrm{means}\pm SD$. The statistically significant difference (using Student’s *t*-test) is indicated by: ***, *P*<0.001.


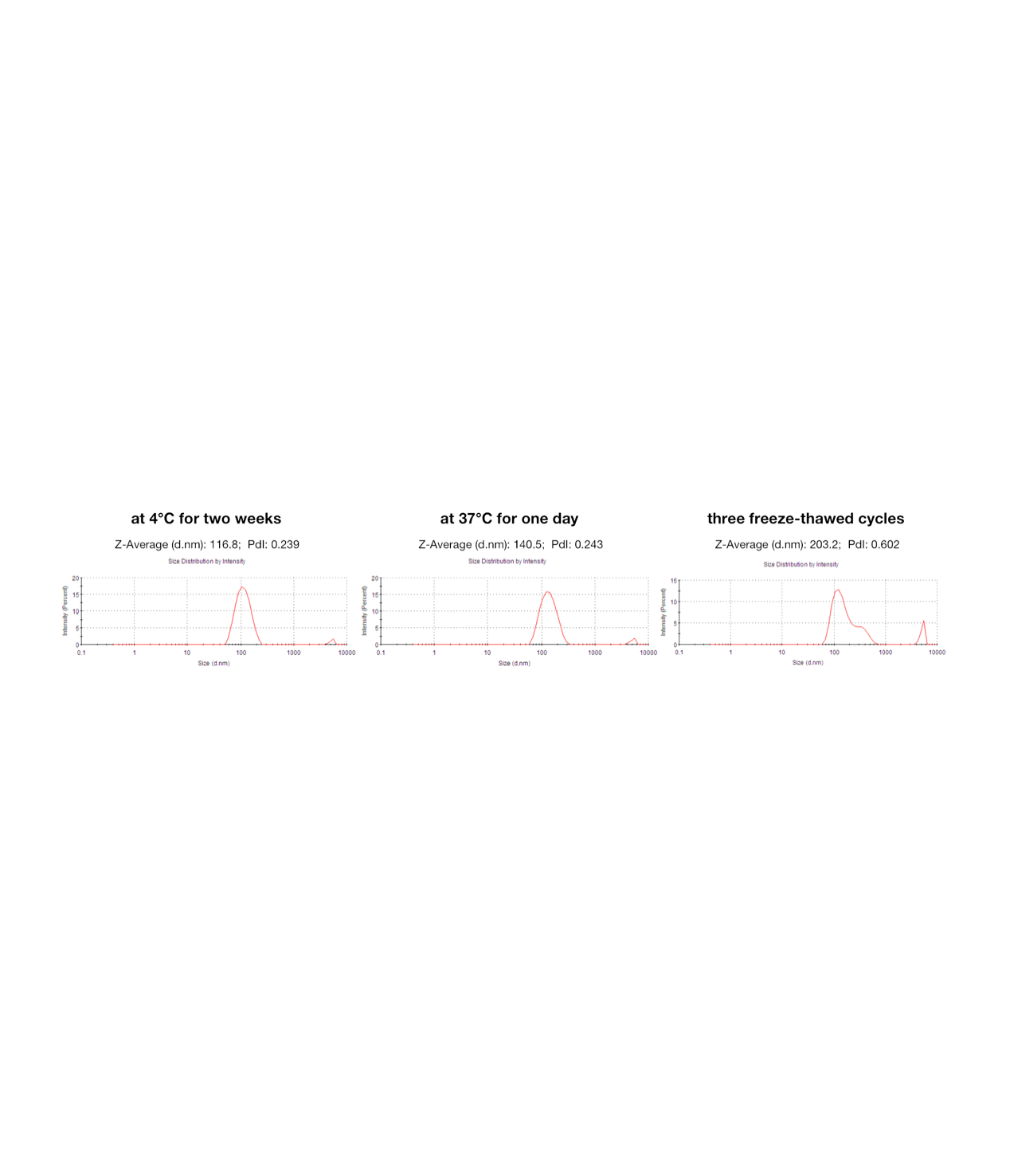


**Figure S4.** Analysis of the VLP stability of 52L1m4CS4 stored at 4℃ for two weeks, at 37℃ for one day, or underwent three freeze-thaw cycles by DLS.

## Supplementary Tables

**Table S1.** Primers used for the synthesis of *6L1CS1* to *6L1CS6* genes

| Primer | Sequence (5’ to 3’) |
| --- | --- |
| F | GGAATTCGCCGCCACCATGTGGCGT |
| CS1-R | R1: CACGCCGGTTGCGATGCTGGATGAGCCTCGGTAGCCGGACTG  R2: AGGAGCGGCGCTAGCCTTGCTGACGGCGGGTCCTGCCACGCCGGTTGCGATGCT  R3: GCTCTAGAATTAACGCTTTGTCCCAGCTCCAGCTCCACTAGGAGCGGCGCTAGCCTTGC |
| CS2-R | R1: CCCACGCCGGTTGCGATGCTGGATCGGCCTCCGTAGCCGGACTGGAGCAGGAATTTCCT  R2: GCAGGAGCGGCGCTAGCTCCGCTGACGGCGGGTGCTCCCACGCCGGTTGCGATGCTGGA  R3: GCTCTAGAATTAACGCTTTGTCCCAGCTGCGGCTCCTGCAGGAGCGGCGCTAGCTCCGC |
| CS3-R | R1: CACGCCGGTTCGGATGCTGGATGAGCCTCGGTAGCCGGACTG  R2: AGGAGCGGCGCTAGCCTTGCTGACGGCGGGTCCTGCCACGCCGGTTCGGATGCT  R3: GCTCTAGAATTAACGCTTTGTCCCAGCTCTAGCTCCATCAGGAGCGGCGCTAGCCTTGC |
| CS4-R | R1: CACGCCGGTTCGGATGCTGGATGAGCCTCGGTAGCCGGACTG  R2: AGGAGCGGCGCTAGCCTTGCTGACGGCGGGACTTGCCACGCCGGTTCGGATGCT  R3: GCTCTAGAATTAACGCTTTGTATCAGCTCTAGCTCCATCAGGAGCGGCGCTAGCCTTGC |
| CS5-R | R1: CACGCCGGTTCGGATGCTGGATGAGCCTCGGTAGCCGGACTG  R2: AGGAGCGGCGCTAGCCTTGCTGACGGCGGGTCCTGCCACGCCGGTTCGGATGCT  R3: GCTCTAGAATTAACGCTTTGTATCAGCTCTAGCTCCATCAGGAGCGGCGCTAGCCTTGC |
| CS6-R | R1: CACGCCGGTTCGGATGCTGGATGAGCCTCGGTAGCCGGACTG  R2: AGGAGCGGCGCTAGCCTTGCTGACGGCGGGACTTGCCACGCCGGTTCGGATGCT  R3: GCTCTAGAATTAACGCTTTGTCCCAGCTCCAGCTCCATCAGGAGCGGCGCTAGCCTTGC |

**Table S2.** Primers used for the synthesis of *11L1CS1* to *11L1CS5* genes

| Primer | Sequence (5’ to 3’) |
| --- | --- |
| F | CGGATCCGCCGCCACCATGTG |
| CS1-R | R1: GGCGGGACTTCCGATGCCGGTCCTGGCGCTGGTGTCGCCCCTGTAGCCGGACTGGAG  R2: CTGCTTCCGTCAGGAGCGGTGCTAGGTTTGCTGACGGCGGGACTTCCGATGCCGGT  R3: CCCAAGCTTATTATTTCTTTGTGTCTGTCCTGCTTCCGTCAGGAGCGGT |
| CS2-R | R1: GGCGGGACTTCCGATGTCGGTCCTGGCGCTGGTTCCGCCCCTGTAGCCGGACTGGAG  R2: CTGCTTCCGTCAGGAGCGGTGCTAGGTTTGCTGACGGCGGGACTTCCGATGTCGGT  R3: CCCAAGCTTATTATTTCTTTGTGTCTGTCCTGCTTCCGTCAGGAGCGGT |
| CS3-R | R1: GGCGGGACTTCCGATGCCGGTCCTGGCGCTGGTTCCGCCCCTGTAGCCGGACTGGAG  R2: CTGCTTCCGTCAGGAGCGGTGCTAGGTTTGCTGACGGCGGGACTTCCGATGCCGGT  R3: CCCAAGCTTATTATTTCTTTGTGTCTGTCCTGCTTCCGTCAGGAGCGGT |
| CS4-R | R1: GGCGGGACTTCCGATGCCGGTCCTGGCGCTGGTTCCGCCCCTGTAGCCGGACTGGAG  R2: CCGCTTCCGCTAGGAGCGGTGCTAGGTTTGCTGACGGCGGGACTTCCGATGCCGGT  R3: CCCAAGCTTATTATTTCTTTGTGTCTGTCCTGCTGTCTCCAGGAGCGGT |
| CS5-R | R1: GGCGGGACTTCCGATGCCGGTCCTGGCGCTGGTTCCGCCCCTGTAGCCGGACTGGAG  R2: CTGCTGTCTCCAGGAGCGGTGCTAGGTTTGCTGACGGCGGGACTTCCGATGCCGGT  R3: CCCAAGCTTATTATTTCTTTGTGTCTGTCCTGCTGTCTCCAGGAGCGGT |

**Table S3.** Primers used for the synthesis of *52L1ΔC* genes with point mutations

| Primer | Sequence (5’ to 3’) |
| --- | --- |
| ΔC-F | CGCGGATCCGCCGCCACCATG |
| ΔC-R | CGCGAATTCCTTACAGTTTAGGTCTAGCTTG |
| N184T-F | CCAGGAGACTGCCCACCTTTGCAGCT |
| N184T-R | GGGCAGTCTCCTGGGGTTCCTGAGTTATTGT |
| Q281K-F | GGTTCCAACAGCGGTAACACAGCC |
| Q281K-R | ACCGCTGTTGGAACCCTTAATATAAAGGTCTC |
| S357D-F | ACATACAAAAACGAGAATTTCAAGGAATACTTGCGT |
| S357D-R | GAAATTCTCGTTTTTGTATGTGTCTTCCTTCTTCACCTC |
| D447E-F | TACATGTTTTGGGAAGTGGATCTCAAAGAGAAG |
| D447E-R | CCACTTCCCAAAACATGTACTCCTTAAGTGGGTCCTC |

**Table S4.** Primers used for the synthesis of *52L1ΔCm4* genes with N-terminal truncations

| Primer | Sequence (5’ to 3’) |
| --- | --- |
| ΔN2-F | CGCGGATCCGCCGCCACCATGGTGTGGCGTCCTTCCGAG |
| ΔN4-F | CGCGGATCCGCCGCCACCATGCGTCCTTCCGAGGCTACTG |
| ΔN5-F | CGCGGATCCGCCGCCACCATGCCTTCCGAGGCTACTGTG |
| ΔN8-F | CGCGGATCCGCCGCCACCATGGCTACTGTGTACTTGCCTC |
| ΔN10-F | CGCGGATCCGCCGCCACCATGGTGTACTTGCCTCCAGTACCT |
| ΔN13-F | CGCGGATCCGCCGCCACCATGCCTCCAGTACCTGTTTCTAAAGTG |
| ΔN15-F | CGCGGATCCGCCGCCACCATGGTACCTGTTTCTAAAGTGGTCTCC |
| ΔN18-F | CGCGGATCCGCCGCCACCATGTCTAAAGTGGTCTCCACTGATGAA |
| ΔN20-F | CGCGGATCCGCCGCCACCATGGTGGTCTCCACTGATGAATACGTCTCA |
| R | CCGGAATTCCTTACAGTTTAGGTCTAGCTTGCAGTC |

**Table S5.** Primers used for the synthesis of *52L1m4CS1* to *52L1m4CS9* genes

| Primer | Sequence (5’ to 3’) |
| --- | --- |
| F | CGCGGATCCGCCGCCACCATG |
| CS1-R | R1: CCGGAATTCCTTACAGTCCTGCTTGCAAGAG  R2: GAGCGCTCGATGCAGGACCTTTCAGTCCAGGACGAGCTTGCAGTCCTGCTTGCAAGAGG  R3: CTTAGCGTCCCACTCCCGAGCCGTCCGTCGACGTTCTAGGAGCGCTCGATGCAGGAC  R4: CCGGAATTCCTTAGCGTCCCACTCCCG |
| CS2-R | R1: CCGGAATTCCTTACAGTCCTGCTTGCAAGAG  R2: GAGCGCTCGATGCAGGACCTTTCAGTCCAGGACGAGCTTGCAGTCCTGCTTGCAAGAGG  R3: CTTAGCCGTCCACTCCCGAGCCGTCCGTCGACGTTCTAGGAGCGCTCGATGCAGGAC  R4: CCGGAATTCCTTAGCCGTCCACTCCCG |
| CS3-R | R1: CCGGAATTCCTTACAGTCCTGCTTGCAAGAG  R2: GAGCGCTCGATGCAGGCGATCCCAGTCCAGGACGAGCTTGCAGTCCTGCTTGCAAGAGG  R3: CTTAGCGTTTCACTCCCGAGCCGTCCGTCGACGTTCTAGGAGCGCTCGATGCAGGC  R4: CCGGAATTCCTTAGCGTTTCACTCCCG |
| CS4-R | R1: CCGGAATTCCTTACAGTCCTGCTTGCAAGAG  R2: GAGCGCTCGATGCAGGCGATCCCAGTCCAGGACGAGCTTGCAGTCCTGCTTGCAAGAGG  R3: CTTAGCGGTCCACTCCCGAGCCGTCCGTCGACGTTCTAGGAGCGCTCGATGCAGGC  R4: CCGGAATTCCTTAGCGGTCCACTCCCG |
| CS5-R | R1: GGTTGAAGTCGCGGGTGCGGAAGAGGCAGGACCAGCCAGT TTAGGTCTAGCTTGCAGTC  R2: CCGGAATTCCTTACGACCCAACTCCGCCGGCAGCGGTTGA AGTCGCGGGTGC |
| CS6-R | R1: GGTTGAAGTACCGGGTGCGGAAGAGGCAGGAGCTTCCAGT TTAGGTCTAGCTTGCAGTC  R2: CCGGAATTCCTTATCCAGCAACCGCTTTCGAGCCGGTTGA AGTACCGGGTGC |
| CS7-R | R1: GGTTGAGGTAGCTGGAGCTGAGGAAGCAGGACCAGCCAGTTTAGGTCTAGCTTGCAGTC  R2: CCGGAATTCCTTAGCGCTTCACACCAGAACCGTCGGTTGAGGTAGCTGGAGCTG |
| CS8-R | R1: GGTTGAGGTACGTGGAGCTGAGGAAGCAGGACCAGCCAGTTTAGGTCTAGCTTGCAGTC  R2: CCGGAATTCCTTAGCGCTTCACACCAGAACCGTCGGTTGAGGTACGTGGAGCTG |
| CS9-R | R1: CCGGAATTCCTTACAGTCCTGCTTGCAAGAG  R2: GCTCGAGGCAGGACCCGACAAGCCAGGACCCGCTTGCAGTCCTGCTTGCAAGAGG  R3: GAGCCACCCGTCGACGTTCTAGGGGCGCTCGAGGCAGGACCCGAC  R4: CCGGAATTCCTTAGCTACCCACGGCCGAGCCACCCGTCGACGTTC |

**Table S6.** Primers used for the synthesis of *52L1m4ΔN13CS1* to *52L1m4ΔN13CS3* genes

| Primer | Sequence (5’ to 3’) |
| --- | --- |
| F | CGCGGATCCGCCGCCACCATGCCTCCAGTACCTGTTTCTAAAGTG |
| CS1-R | R1: CCGGAATTCCTTACAGTCCTGCTTGCAAGAG  R2: GCTCGAGGCAGGACCCGACAAGCCAGGTCTCGCTTGCAGTCCTGCTTGCAAGAGG  R3: GAGCCACCCGTCGACGTTCTAGGGGCGCTCGAGGCAGGACCCGAC  R4: CCGGAATTCCTTAGCTACCCACGGCCGAGCCACCCGTCGACGTTC |
| CS2-R | R1: CCGGAATTCCTTACAGTCCTGCTTGCAAGAG  R2: GCTCGAGGCAGGACCCGACAAGCCAGGACCCGCTTGCAGTCCTGCTTGCAAGAGG  R3: GAGCCACCCGTCGACGTTCTAGGGGCGCTCGAGGCAGGACCCGAC  R4: CCGGAATTCCTTAGCTACCCACGGCCGAGCCACCCGTCGACGTTC |
| CS3-R | R1: CGTCGACGTGGCAGGGGCGCTCGAGGCAGGACCGGCCAGTTTAGGTCTAGCTTGCAGTC  R2: CCGGAATTCCTTAGCTACCCACGCCTCCCGCAGCCGTCGACGTGGCAGGGG |

**Table S7.** Primers used for the synthesis of *16L1-33L2 CS1* to *16L1-33L2 CS6* genes

| Primer | Sequence (5’ to 3’) |
| --- | --- |
| F | CCGGAATTCCTTACAGTTTAGGTCTAGCTTGCAGTC |
| CS1-R | R1: TGCACCGGAAGCGCCCAAAGTGAAGGAAGGACCAGCCTTGAGACCAGCTTGCAACAGG  R2: CCGCCAGCTGTGGTACTAGTGGATGACGTTGTGGGAGTTGCACCGGAAGCGCCCAAAG  R3: CCGGAATTCCTTACAAAGAGCCAGCGCTACCGCCAGCTGTGGTACTAG |
| CS2-R | R1: TGCACCGGAAGCGCCCAAAGTGAAGGAAGGACCAGCTCCGAGACCAGCTTGCAACAGG  R2: CCGCCAGCTGTGGTACTAGTGGATGACGTTGTGGGAGTTGCACCGGAAGCGCCCAAAG  R3: CCGGAATTCCTTACAAAGAGCCAGCGCTACCGCCAGCTGTGGTACTAG |
| CS3-R | R1: CTTTCGATCCACCCAGCGTAAACTTGGGACCGGCCTTGAGACCAGCTTGCAACAGG  R2: CGTCTGCCGTCGTAGACGTATCCGAAGTCGTAGGCGTAGCTTTCGATCCACCCAGCG  R3: CCGGAATTCCTTACAATTTACCGTCCGAGCCGTCTGCCGTCGTAGAC |
| CS4-R | R1: CTTTCGATCCACCCAGCGTAAACTTGGGACCGGCCTTGAGACCAGCTTGCAACAGG  R2: CCTGCCGTCGTAGACGTATCCGAAGTCGTAGGCGTAGCTTTCGATCCACCCAGCG  R3: CCGGAATTCCTTACAATTTCGAGCCTTTCGAGCCTGCCGTCGTAGACGTATC |
| CS5-R | R1: CTTTCGATCCACCCAGCGTAAACTTGGGACCGGCCTTGAGACCAGCTTGCAACAGG  R2: GTCTGCCGTCGTAGACGTCGACGAAGTCGTAGGCGTAGCTTTCGATCCACCCAGCG  R3: CCGGAATTCCTTACAATTTACCGTCCGAGCCGTCTGCCGTCGTAGACGTC |
| CS6-R | R1: CTTTCGATCCACCCAGCGTAAACTTGGGACCGGCCTTGAGACCAGCTTGCAACAGG  R2: CCTGCCGTCGTAGACGTCGACGAAGTCGTAGGCGTAGCTTTCGATCCACCCAGCG  R3: CCGGAATTCCTTACAATTTCGAGCCTTTCGAGCCTGCCGTCGTAGACGTC |

**Table S8.** Primers used for the synthesis of *58L1-16L2 CS1* to *58L1-16L2 CS3* genes

| Primer | Sequence (5’ to 3’) |
| --- | --- |
| F | CCGGAATTCCTTACAGTTTAGGTCTAGCTTGCAGTC |
| CS1-R | R1: GTCGTAGGGGCCGAGCCGGCCAACGAAGGGCCGGCTTTCAAGCCGCTCTGCAGCAGG  R2: CTGCCACCCGTCGACGGCGCCGAGGTCGTAGGGGCCGAGCCG  R3: GCTCTAGACTTAGCTACCCACGGCGCTGCCACCCGTCGACGG |
| CS2-R | R1: GTCGTAGGGGCCGAGCCGGCCAACGAAGGGCCGGCACCCAAGCCGCTCTGCAGCAGG  R2: CTGCCACCCGTCGACGGCGCCGAGGTCGTAGGGGCCGAGCCG  R3: GCTCTAGACTTAGCTACCCACGGCGCTGCCACCCGTCGACGG |
| CS3-R | R1: GTCGTAGGGGCCGAGCCGGCCAACGAAGGGCCGGCTTTCAAGCCGCTCTGCAGCAGG  R2: CTGCCACCCGTCGACGGCGCTCTGGTCGTAGGGGCCGAGCCG  R3: GCTCTAGACTTAGCTACCCACGGCGCTGCCACCCGTCGACGG |

**Table S9.** Minimum free energy of mRNA secondary structure

|  | Minimum Free Energy  (kcal/mol) |
| --- | --- |
| 6L1 | -466.0 |
| 6L1CS1 | -483.8 |
| 6L1CS2 | -482.4 |
| 6L1CS3 | -477.3 |
| 6L1CS4 | -471.2 |
| 6L1CS5 | -473.6 |
| 6L1CS6 | -476.3 |
| 11L1 | -488.8 |
| 11L1CS1 | -495.1 |
| 11L1CS2 | -494.0 |
| 11L1CS3 | -495.2 |
| 11L1CS4 | -496.5 |
| 11L1CS5 | -496.5 |
| 52L1m4ΔC | -428.4 |
| 52L1m4CS1 | -466.3 |
| 52L1m4CS2 | -461.6 |
| 52L1m4CS3 | -457.0 |
| 52L1m4CS4 | -459.0 |
| 52L1m4CS5 | -460.0 |
| 52L1m4CS6 | -450.3 |
| 52L1m4CS7 | -458.0 |
| 52L1m4CS8 | -455.3 |
| 52L1m4CS9 | -465.0 |
| 52L1m4ΔN13 | -416.2 |
| 52L1m4ΔN13CS1 | -449.9 |
| 52L1m4ΔN13CS2 | -449.9 |
| 52L1m4ΔN13CS3 | -450.0 |
| 16L1-33L2 | -450.3 |
| 16L1-33L2 CS1 | -485.1 |
| 16L1-33L2 CS2 | -486.4 |
| 16L1-33L2 CS3 | -497.2 |
| 16L1-33L2 CS4 | -492.7 |
| 16L1-33L2 CS5 | -498.2 |
| 16L1-33L2 CS6 | -489.7 |
| 58L1-16L2 | -444.1 |
| 58L1-16L2 CS1 | -491.6 |
| 58L1-16L2 CS2 | -493.8 |
| 58L1-16L2 CS3 | -490.8 |

# Supplementary Reference

Wang, Z., Zhang, T., and Xu, X. (2022). Combined truncations at both N- and C-terminus of human papillomavirus type 58 L1 enhanced the yield of virus-like particles produced in a baculovirus system. *J. Virol. Methods* 301, 114403. doi: 10.1016/j.jviromet.2021.114403.
