# Supplementary figures and images for: A novel C-terminal modification method enhanced the yield of human papillomavirus L1 or chimeric L1-L2 virus-like particles in the baculovirus system
Source: Front Bioeng Biotechnol. 2023 Jan 5;10:1073892. doi: 10.3389/fbioe.2022.1073892 (PMC9849392; doi:10.3389/fbioe.2022.1073892)

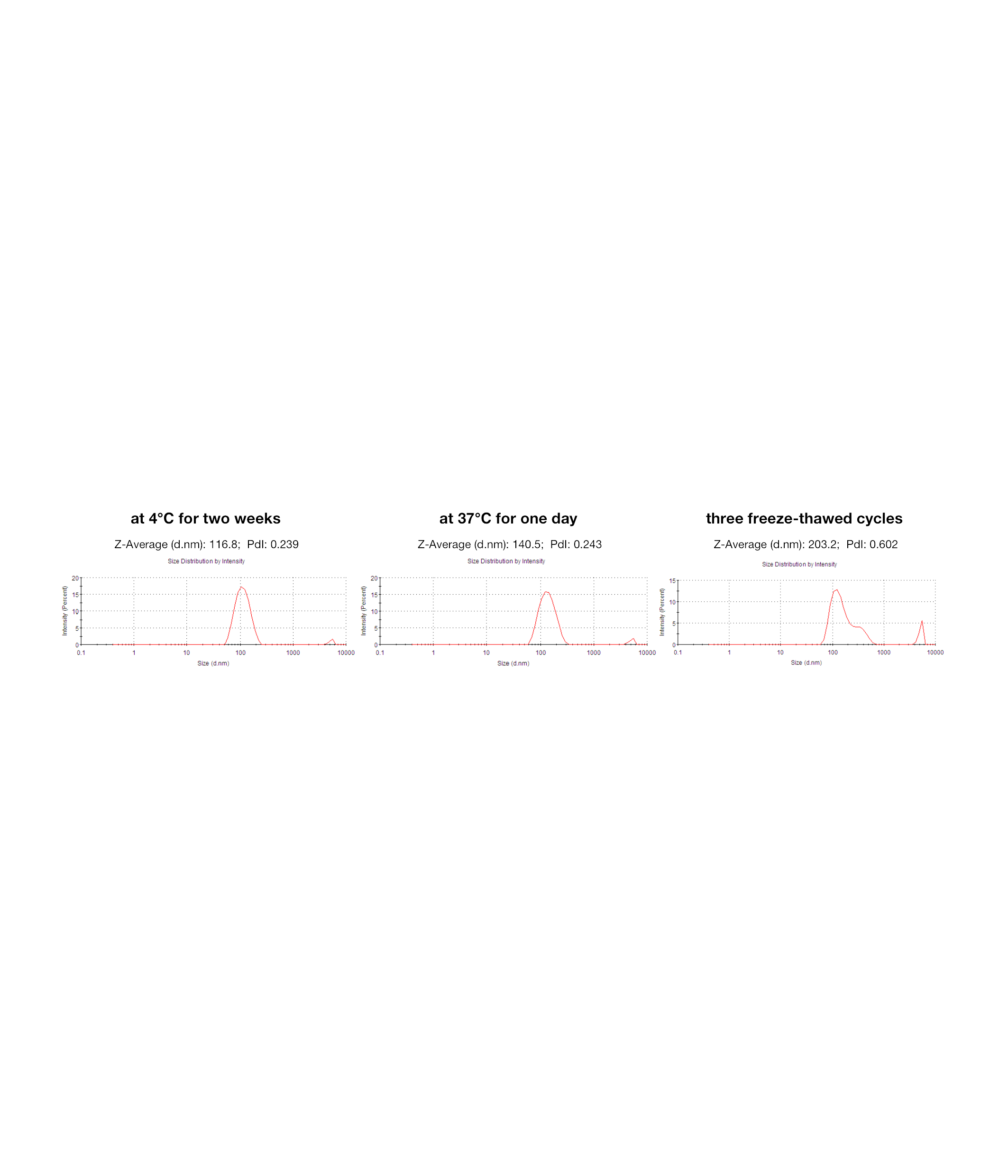

Supplement: Supplementary file 2 [file DataSheet1.ZIP › Supplementary figures/Fig S4.tif]

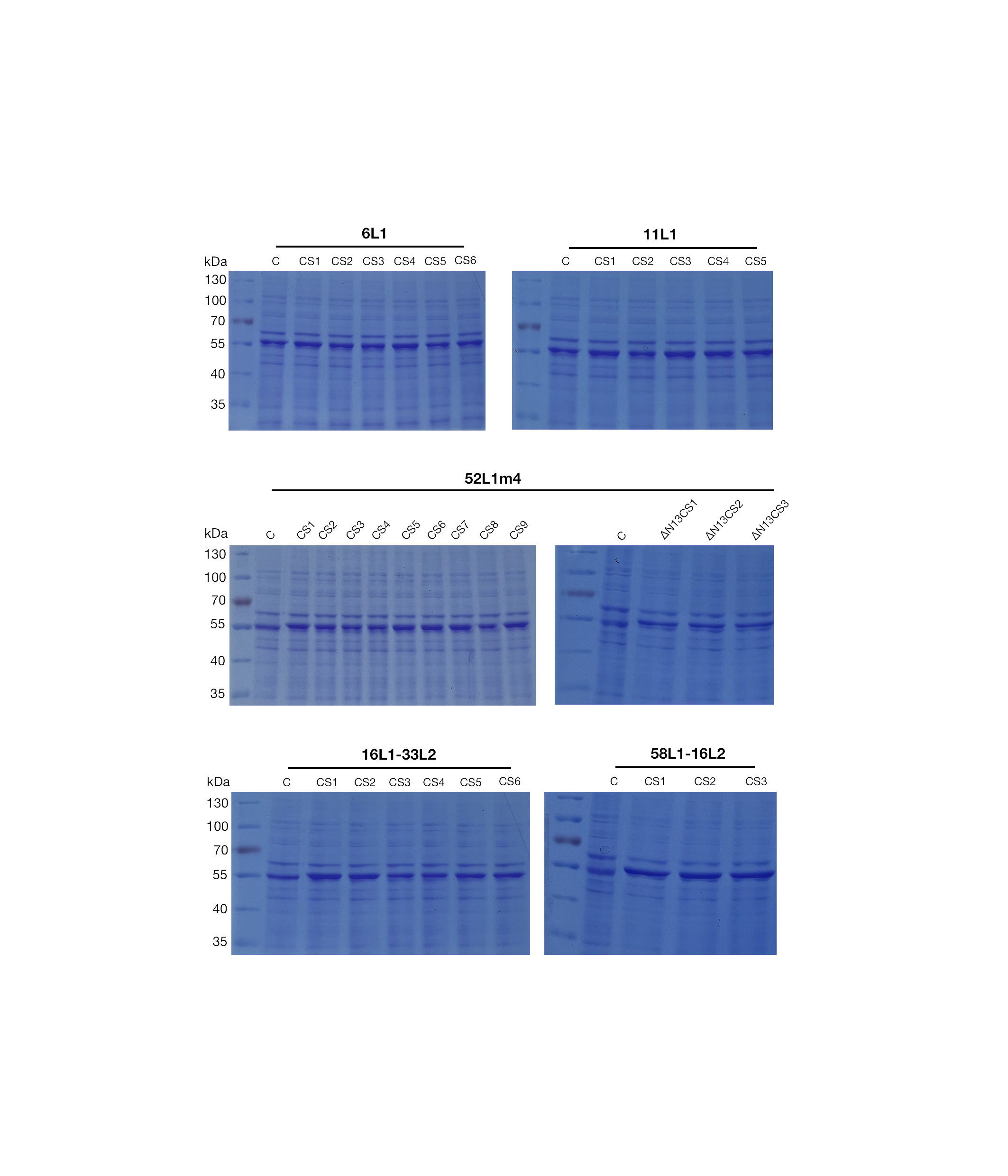

Supplement: Supplementary file 2 [file DataSheet1.ZIP › Supplementary figures/Fig S1.tif]

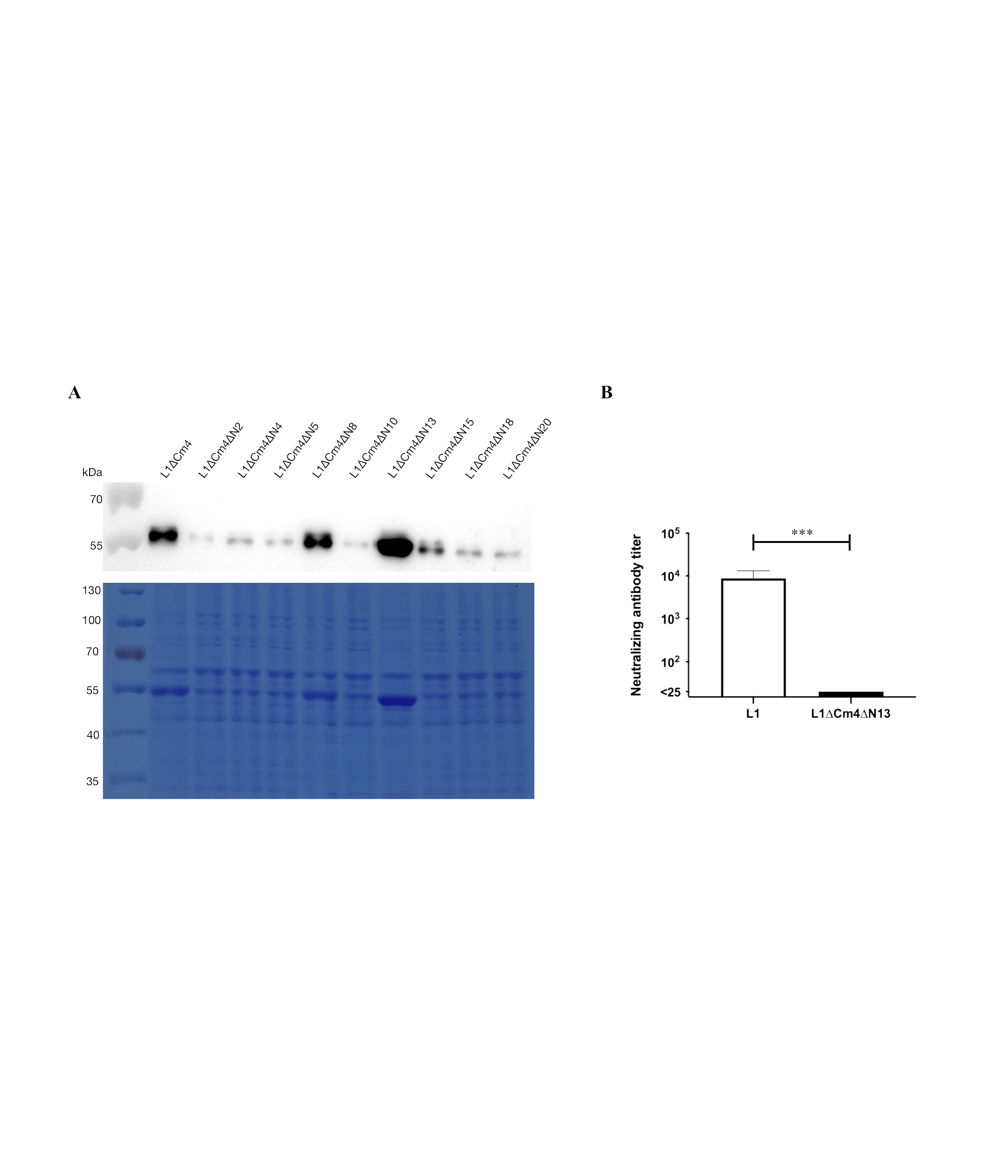

Supplement: Supplementary file 2 [file DataSheet1.ZIP › Supplementary figures/Fig S3.tif]

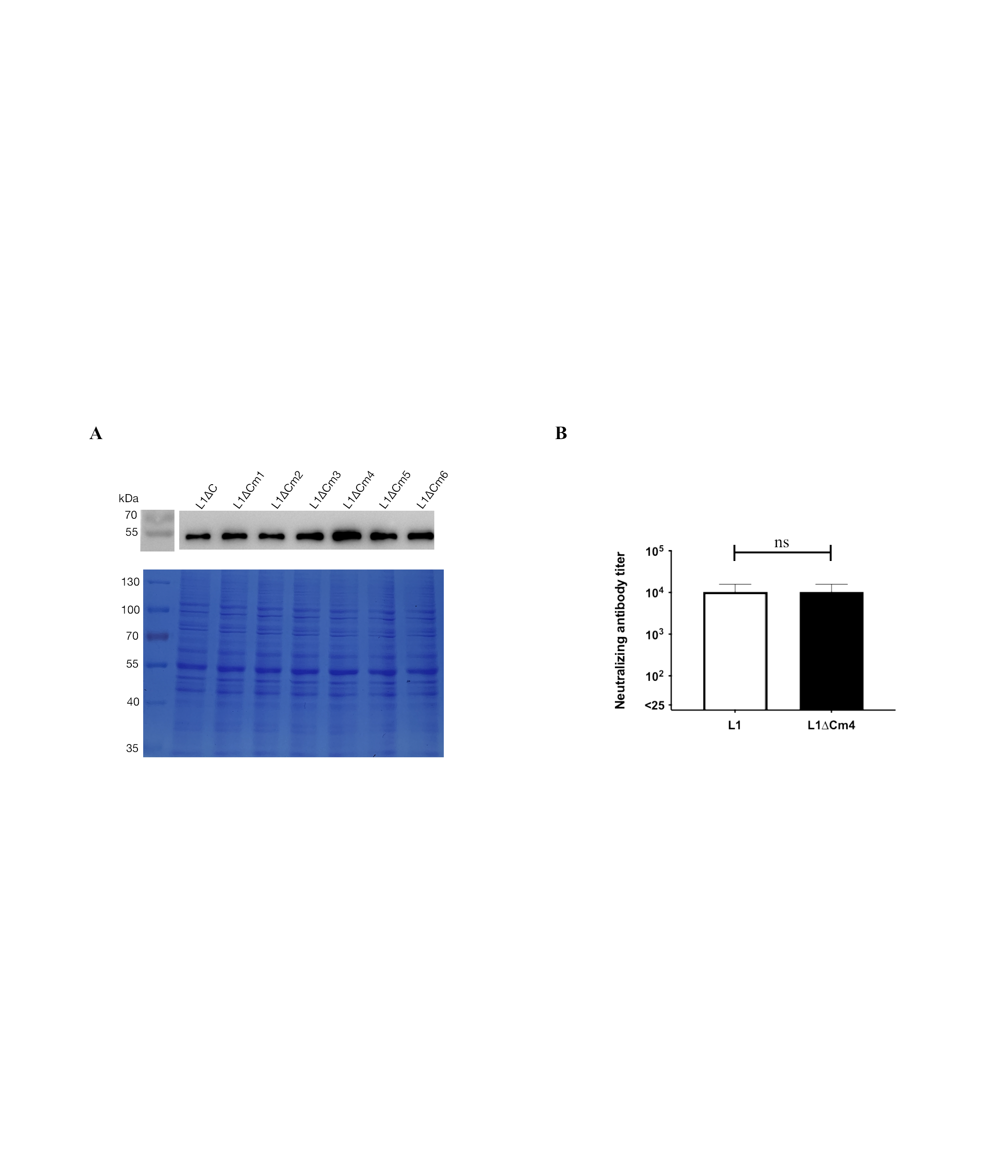

Supplement: Supplementary file 2 [file DataSheet1.ZIP › Supplementary figures/Fig S2.tif]

**raw data (manuscript ID: 1073892)**

https://www.jianguoyun.com/p/DTx_pWQQ0o2ECxi7quAEIAA


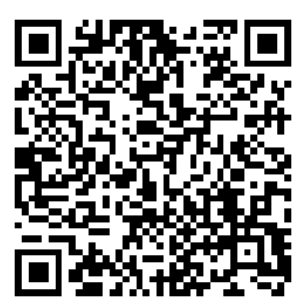

Supplement: Supplementary file 3 [file DataSheet2.docx]
